# Chemical Analysis and Antioxidant Activities of Resin Fractions from *Pistacia lentiscus* L. *var. Chia* in Neuroblastoma SH-SY5Y Cells

**DOI:** 10.3390/molecules30050997

**Published:** 2025-02-21

**Authors:** Achilleas Georgantopoulos, Foteini D. Kalousi, Federica Pollastro, Ioannis Tsialtas, Natasa P. Kalogiouri, Anna-Maria G. Psarra

**Affiliations:** 1 Department of Biochemistry and Biotechnology, University of Thessaly, Biopolis, 41500 Larissa, Greece; ageorgant@uth.gr (A.G.); fokalous@uth.gr (F.D.K.); tsialtasj@gmail.com (I.T.); 2 Department of Pharmaceutical, University of Eastern Piedmont, 28100 Novara, Italy; federica.pollastro@uniupo.it; 3 Laboratory of Analytical Chemistry, School of Chemistry, Aristotle University of Thessaloniki, 54124 Thessaloniki, Greece; kalogiourin@chem.auth.gr

**Keywords:** Chios mastiha, *Pistacia lentiscus*, antioxidant activity, SH-SY5Y cells, ROS, glucocorticoid receptor, PPARα, neuroprotection

## Abstract

Chios mastiha is the natural aromatic resin of *Pistacia lentiscus* L. *var. Chia*, *Anacardiaceae,* which is exclusively cultivated in the southern part of the Greek island of Chios. Chios mastiha (*P. lenticonus*/Chios mastiha) is well-known for its distinctive taste and aroma and has been known since ancient times due to its healing properties in gastrointestinal and inflammatory disorders and because of its anti-bacterial and anti-fungal activities. In this study, the chemical composition, applying LC-QTOF-MS/MS analysis, and the antioxidant activities of three different polarity *P. lenticonus*/Chios mastiha fractions, apolar, medium polar, and polar, were characterized in human neuroblastoma SH-SY5Y cells. Chemical analysis of the fractions unveiled new components of *P. lenticonus*/Chios mastiha, mainly fatty acids compounds, known for their antioxidant activity and regulatory effects on lipid metabolism. By applying the MTT assay and confocal microscopy analysis, we showed that *P. lenticonus*/Chios mastiha fractions, especially the apolar and medium polar fractions, enriched in triterpenes and fatty acids, caused suppression of the H_2_O_2_-induced reduction in cell viability, ROS production, and depolarization of the mitochondrial membrane potential, in SH-SY5Y cells. Moreover, Western blot analysis revealed that apolar fraction, enriched in fatty acids, induced expression of the PPARα, which is well-known for its antioxidant activities and its crucial role in lipid metabolism. Induction of PPARα, a GR target gene, was also accompanied by an increase in GR protein levels. Enhanced antioxidant activities of the apolar fraction may be correlated with its chemical composition, enriched in fatty acids and triterpenoids. Thus, our results indicate the neuroprotective actions of *P. lenticonus*/Chios mastiha fractions, highlighting their potential application as neuroprotective agents in neurodegenerative diseases.

## 1. Introduction

*Pistacia lentiscus* L. *var. Chia* (Mastic Tree) belongs to the *Anacardiaceae* family. Even though *P. lentiscus* is distributed throughout the Mediterranean, the Chia variety is exclusively cultivated on the Greek island of Chios, particularly in the southern part of the island, due to the climatic and soil conditions that prevail there. Chios mastiha, or Chios mastic gum (*P. lenticonus*/Chios mastiha), is the natural aromatic resin derived from the trunk and branches of the *Pistacia lentiscus* L. *var. Chia*. *P. lenticonus*/Chios mastiha has been harvested for over 2500 years; it is widely used as a food flavoring additive and in beverages and it has many applications in producing natural chewing gum, skin-, dental-, and cosmetic products. Most importantly, *P. lenticonus*/Chios mastiha has been known since ancient times for its healing properties in gastrointestinal and inflammatory disorders [1]. In 2015 *P. lenticonus*/Chios mastiha was awarded by the European Medicine Agency as a natural medicine for skin inflammation and dyspeptic disorders [1,2]. Moreover, anti-cancer [3,4,5] and cardiovascular protective activities [6,7] of *P. lenticonus*/Chios mastiha have been proposed. These actions are attributed to the anti-bacterial, anti-fungal [8] anti-inflammatory [9,10,11,12] antilipemic, antioxidant, and apoptotic [7,13,14] activities of *P. lenticonus*/Chios mastiha and its essential oil.

*P. lenticonus*/Chios mastiha’s therapeutic actions on gastrointestinal and inflammatory disorders, namely inflammatory bowel diseases, are associated with the *P. lenticonus*/Chios mastiha’s protective anti-inflammatory effects on intestinal epithelial cells [11,15]. Anti-inflammatory actions of *P. lenticonus*/Chios mastiha are also beneficial for other cell types [9,10,16], including cardiovascular cells, where the antioxidant activities of *P. lenticonus*/Chios mastiha are suggested to play a crucial role. Specifically, it has been shown that treatment with *P. lenticonus*/Chios mastiha improves the total antioxidant status of patients with nonalcoholic fatty liver disease (NAFLD) [17] and the antioxidant activity of oxidized low-density lipoprotein (LDL) in peripheral blood mononuclear cell (PBMC) cells, suppressing the main causative factor of atherosclerosis [6]. Increased reactive oxygen species (ROS) levels are responsible for many pathological conditions primarily for neurodegenerative diseases [18] and cancer. In this context, the apoptotic and antiproliferative properties of *P. lenticonus*/Chios mastiha in neuroblastoma SH-SY5Y and SK-N-BE cells [19] have been reported. Moreover, due to the fundamental role of mitochondria in ROS production and energy metabolism, maintenance of mitochondria functionality is crucial for both cancer and neurodegenerative diseases [20]. Thus, further investigation of the neuroprotective actions of *P. lenticonus*/Chios mastiha and its potential anti-cancer activity on neuroblastoma cells, with emphasis on its effect on the preservation of mitochondrial functionality could be of great importance.

Biological activities of *P. lenticonus*/Chios mastiha are associated with its chemical composition. To this point, chemical analyses of *P. lenticonus*/Chios mastiha, applying gas spectrometry, mass spectrometry, and NMR analysis, have revealed that the sticky texture of the resin is attributed to a component named β-polymyrcene, which constitutes approximately 25–30% of the total composition. The rest, 65–70% of the *P. lenticonus*/Chios mastiha is enriched in terpenoids [21,22,23]. Thus, terpenoids such as masticadienonic, isomasticadienonic acid, moronate, oleanolate, and oleanonate, have been identified in the acidic fraction of *P. lenticonus*/Chios mastiha. Moreover, triterpenic compounds like oleanolic aldehyde, 28-norolean-17-en-3-one, β-amyrone, isomasticadienolic aldehyde, tirucallol, and dammaradienone, lupeol, 24Z-masticadienonic acid methyl ester, 24Z-isomasticadienonic acid methyl ester keto-oleanolic aldehyde, and oleanolic aldehyde, have been detected in neutral fractions or medium polar fractions of *P. lenticonus*/Chios mastiha [12,14]. In addition, other phenolic and flavonoid compounds such as gallic acid, caffeic acid, α-terpinolene, linalool, benzoic acid, phenylacetic acid, and tyrosol have been detected, although in smaller amounts [12,13,21,24,25].

The structural similarity of triterpenoids to the steroid hormone glucocorticoid that acts through its cognate receptor, the glucocorticoid receptor (GR) [26,27,28], indicates that triterpenoids could interact with glucocorticoid signaling, affecting many glucocorticoid-mediated biological actions, including energy metabolism, inflammation, and apoptosis. In this context, we have recently shown that *P. lenticonus*/Chios mastiha essential oil, as well as different polarity fractions from Chios Mastic tree leaves and resin, enriched in triterpenoids, could exert anti-inflammatory actions and regulation of apoptosis [12,14,29], affecting both glucocorticoid receptor activity and protein levels. The association of *P. lenticonus*/Chios mastiha enrichment in triterpenoids and phenol compounds with its anti-inflammatory, anti-microbial, and apoptotic activities is also supported by data in the literature [6,7,30,31,32,33,34,35,36].

In this study, LC-QTOF-MS/MS analysis of three different polarity fractions, apolar (a), medium polar (mp), and polar (p) from *P. lenticonus*/Chios mastiha, diluted in DMSO, was applied with the aim of identifying additional unidentified *P. lenticonus*/Chios mastiha’s components of chemical origin, other than terpenoids and phenolic compounds. In addition, the possible neuroprotective actions of *P. lenticonus*/Chios mastiha’s fractions were evaluated by investigating their possible antioxidant activities, their effect on mitochondrial functionality, and their interference with steroid receptors signaling in human neuroblastoma SH-SY5Y cells. In consequence, assessment of the *P. lenticonus*/Chios mastiha fractions’ effects on the H_2_O_2_-induced reduction in SH-SY5Y cell viability, depolarization of the mitochondrial membrane, ROS production, and regulation of GR and antioxidant factor levels, applying MTT assay, confocal microscopy, and Western blot analysis was performed. To the best of our knowledge, this is the first time that new compounds such as palmitic, stearic, and myristic acids in *P. lenticonus*/Chios mastiha’s different polarity fractions were detected. Notably, the study revealed the presence of 37 compounds in *P. lenticonus*/Chios mastiha’s fractions, identified through suspect and non-target screening. Most importantly, the antioxidant activity of *P. lenticonus*/Chios mastiha fractions in SH-SY5Y cells was revealed, highlighting their potential application as neuroprotective agents.

## 2. Results

### 2.1. Chemical Characterization of the Three Different Polarity Fractions of P. lenticonus/Chios Mastiha

Previous studies on the chemical characterization of *P. lenticonus*/Chios mastiha revealed its enrichment in terpenoid compounds [14,37,38,39]. Although extensive studies have focused on terpenoid compound identification [14,37,38,39,40,41], the *P. lenticonus*/Chios mastiha composition involves unidentified compounds that remain to be characterized. To this effort, HPLC-QTOF-MS/MS analysis was performed in three different polarity *P. lenticonus*/Chios mastiha fractions (ap, mp, p). The analysis revealed a plethora of masses *m/z* (Table 1 and Table 2 and Supplementary data, Tables S1–S3, Figures S1 and S2). The identification of the suspect compounds was carried out according to Karadimou et al. [42].

Twenty-eight compounds were identified through suspect screening (Table 1) and are presented along with their literature-reported biological activities in Supplementary data, Table S1. Non-target identification (Supplementary data, Table S3) was performed using the SCIEX Natural Products Library with a Library Match Score above 50.0, according to Mitsikaris et al. [43]. All the tentative compounds were semi-quantified using the calibration curve of caffeic acid and were expressed as equivalents. Nine compounds were identified through non-target screening (Supplementary data, Table S2). Tentatively identified compounds involve fatty acids (Table 1), whose presence in *P. lenticonus*/Chios mastiha is reported for the first time. Specifically, the enrichment of fatty acids, such as palmitic acid, stearic, myristic, arachidonic, and pentadecanoic acid was observed in the ap fraction (Table 1). The exclusive enrichment of the coniferaldehyde compound in the ap was also tentatively identified. Moreover, to the best of our knowledge, the enrichment of mp and p fractions in compounds such as betulinic acid, euscaphic acid (triterpenoid with antioxidant activity), flavidin, luteolin glucoside, and fatty acids such as α-linolenic acid, ricinoleic acid, crepenynic acid, and gamma-linolenic acid is revealed for the first time (Table 1). In summary, except for oleanolic acid, octyl formate, 6,7-dihydroxylinalool, α-irone, methoxycinnamic, and masticadecanoic acid, which were previously identified as *P. lenticonus*/Chios mastiha components [1,21], the compounds in Table 1 are reported for the first time as *P. lenticonus*/Chios mastiha’s components. Furthermore, nine non-target new compounds were detected in different polarity fractions from *P. lenticonus*/Chios mastiha (Table 2). Interestingly, unequivocal molecular formulas were attributed to approximately 250 detected features (Supplementary Data, Table S3), highlighting *P. lenticonus*/Chios mastiha’s rich composition.

### 2.2. Different Polarity Fractions from P. lenticonus/Chios Mastiha Induced Resistance to H_2_O_2_-Triggered Reduction in Cell Viability of SH-SY5Y Cells

**Figure 1:**
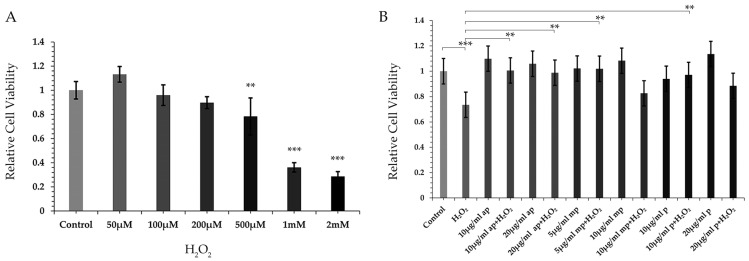
Resistance to H_2_O_2_-induced reduction in neuroblastoma SH-SY5Y cells viability, by the different polarity *P. lenticonus*/Chios mastiha’s fractions. (**A**) Relative cell viability, assessed by MTT assay, of SH-SY5Y cells treated with 50–500 μΜ H_2_O_2_ for 6 h and 1 mM or 2 mM H_2_O_2_ for 3 h. Viability of vehicle-treated (1/1000 *v/v* DMSO) control cells was set as 1. (**B**) Relative cell viability of SH-SY5Y cells pretreated or not with ap, mp, and p *P. lenticonus*/Chios mastiha’s fractions for 24 h and/or not subsequently subjected to 700 μΜ H_2_O_2_ for 6 h. Viability of control (vehicle-treated, 1/1000 *v/v* DMSO) cells was set as 1. Relative cell viability is expressed as the viability of the cells pretreated with various concentrations of the ap, mp, and p fractions compared to the viability of the control cells. Data were analyzed by 1-way ANOVA (Figure 1A) and 2-way ANOVA (Figure 1B), respectively, and are expressed as mean ± SD (*n* = 4, *n* = 5), ** *p* < 0.01 *** *p* < 0.001.

To assess the antioxidant activity and the potential neuroprotective effects of the different polarity fractions from *P. lenticonus*/Chios mastiha, their possible defensive actions on H_2_O_2_-induced reductions in cell viability of SH-SY5Y cells, by applying the MTT assay, were investigated. Firstly, we focused on setting up experimental conditions that lead to approximately 30% H_2_O_2_-induced reduction in the cell viability of SH-SY5Y cells. A moderate (20–30%) and not a severe reduction in cell viability by H_2_O_2_ could allow us to observe any possible protective effects achieved through pretreatment with potential antioxidant factors. Thus, the effects of 50 μΜ to 500 μΜ H_2_O_2_ treatment for 6 h, and 1 mM or 2 mM H_2_O_2_ treatment for 3 h on SH-SY5Y cell viability (Figure 1A) were examined. As shown in Figure 1A, the incubation of SH-SY5Y cells with 500 μΜ H_2_O_2_ for 6 h caused approximately 20% reduction in cell viability, whereas incubation of the cells with 1 mM H_2_O_2_ for 3 h caused more than 60% reduction in cell viability. Thus, incubation of the cells with 700 μΜ H_2_O_2_ for 6 h was considered an optimal condition to study the possible antioxidant activity of the different polarity fractions from *P. lenticonus*/Chios mastiha. Then, the MTT assay was applied in SH-SY5Y cells pretreated with different polarity fractions from *P. lenticonus*/Chios mastiha, at concentrations of 10 and 20 μg/mL for the ap fraction, 5 and 10 μg/mL for the mp fraction, and 10 and 20 μg/mL of p fraction for 24 h, and subsequently subjected to 700 μΜ H_2_O_2_ treatment for an additional 6 h. As shown in Figure 1B, after 24 h of incubation, 10 and 20 μg/mL of ap fraction, 5 and 10 μg/mL of mp fraction, and 10 and 20 μg/mL of p fraction did not cause any statistically significant reduction in cell viability of SH-SY5Y cells. Moreover, as was expected, incubation of SH-SY5Y cells with 700 μΜ H_2_O_2_ for 6 h caused an approximately 30% statistically significant reduction in cell viability compared to control cells. Interestingly, statistically significant resistance to H_2_O_2_-induced reduction in cell viability was observed in cells pre-incubated with 10 μg/mL or 20 μg/mL of ap fraction for 24 h and then subjected to 700 μΜ H_2_O_2_ treatment (for 6 h). Resistance to H_2_O_2_-induced reduction in cell viability was also observed, although to a lower extent, with pretreatment of the cells using 5 μg/mL of the mp fraction and 10 μg/mL of the p fraction.

### 2.3. Protective Effect of the Different Polarity P. lenticonus/Chios Mastiha Fractions on H_2_O_2_-Induced ROS Production in SH-SY5Y Cells

**Figure 2:**
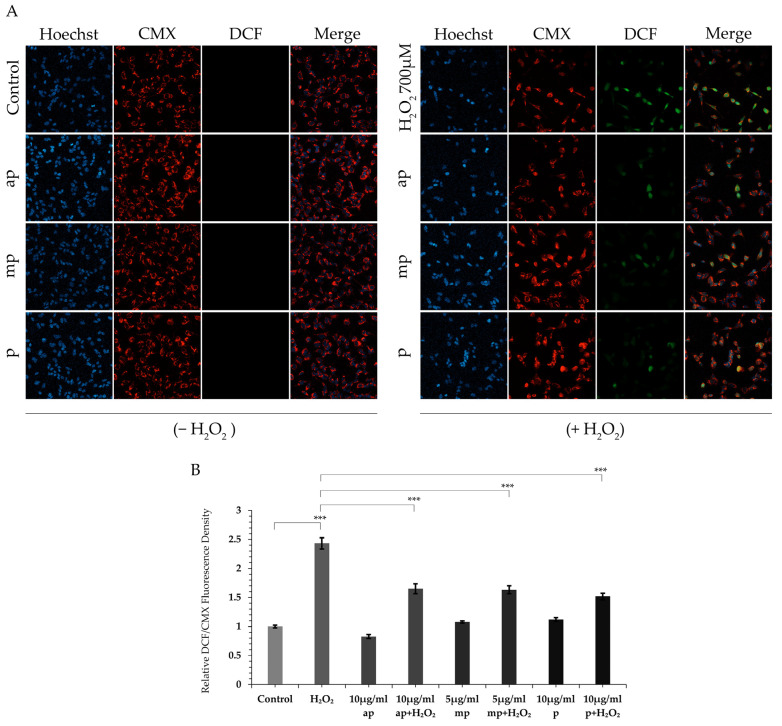
Reduced ROS production in SH-SY5Y cells pretreated with the *P. lenticonus*/Chios mastiha’s fractions and subsequently subjected to H_2_O_2_-induced oxidative stress. (**A**) Representative confocal microscopy single images of SH-SY5Y cells stained with Hoechst-33342 (nuclear staining), MitoTracker CMXRos (mitochondrial staining in living cells, Red), DCF (ROS production dye, Green) upon pretreatment with 10 μg/mL ap, 5 μg/mL mp and 10 μg/mL p *P. lenticonus*/Chios mastiha’s fractions for 24 h and/or not subsequently subjected to 700 μΜ H_2_O_2_ for 6 h. Vehicle-treated, control cells were incubated with 1/1000 *v/v* DMSO. Representative images were taken with Zeiss LSM-800 confocal microscope in 40× objective (**B**) Quantification analysis of DCF staining per CMX staining of single cells. The ratio of DCF/CMX staining in control cells was set as 1. Data were analyzed by 2-way ANOVA (Figure 2) and relative DCF fluorescence density is expressed as mean ± SD (*n* = 60–80), *** *p* < 0.001.

The potential antioxidant activity of the different polarity *P. lenticonus*/Chios mastiha fractions was estimated by measuring ROS production in H_2_O_2_-treated cells. ROS production was evaluated by assessing DCF staining in SH-SY5Y cells pretreated or not with the *P. lenticonus*/Chios mastiha’s fractions and subsequently subjected to H_2_O_2_-induced ROS production. Confocal microscopy images of the DCF dye, an indicator of ROS production, were taken in live SH-SY5Y cells. As shown in Figure 2, treatment of SH-SY5Y cells with 700 μΜ H_2_O_2_ for 6 h caused a statistically significant 2.5-fold increase in DCF staining compared to control cells. Interestingly, pretreatment of SH-SY5Y cells with the *P. lenticonus*/Chios mastiha’s fractions, for 24 h, at the indicated concentrations, provided them with resistance to H_2_O_2_-induced ROS production, leading to a statistically significant decrease in DCF staining compared to that in H_2_O_2_-treated cells. In accordance with the results from the MTT assay, the ap fraction showed the highest antioxidant activity.

### 2.4. Maintenance of the Mitochondrial Functionality by the Different Polarity P. lenticonus/Chios Mastiha Fractions upon Conditions of H_2_O_2_-Induced Oxidative Stress

**Figure 3:**
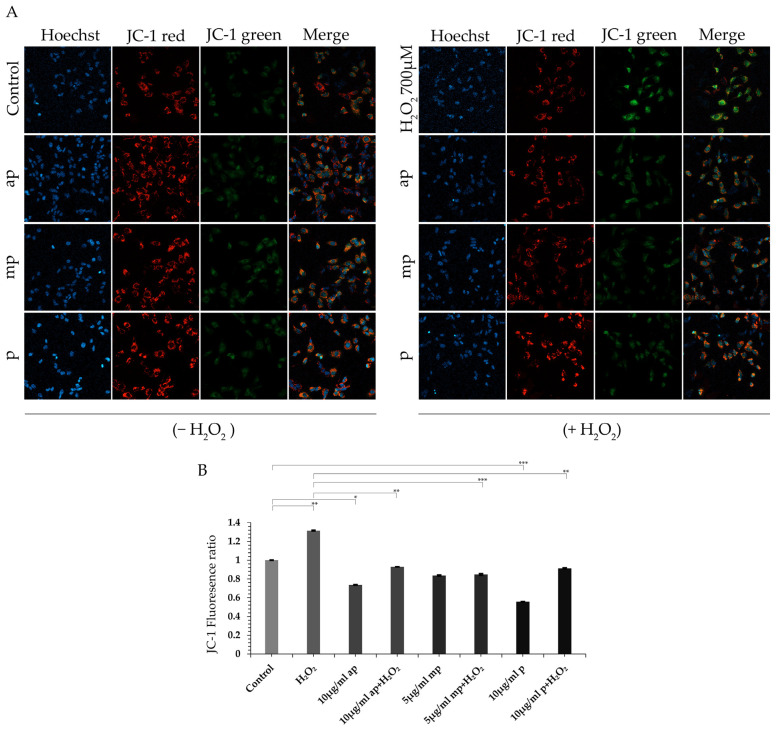
Antioxidant activities of the different polarity fractions of *P. lenticonus*/Chios mastiha’s contribute to the maintenance of the mitochondrial membrane potential in H_2_O_2_-treated SH-SY5Y cells. (**A**) Representative images of JC-1 staining (JC-1 dimers-red aggregates; JC-1 monomers-green fluorescence) and Hoechst-33342 staining (nuclear staining) in human neuroblastoma SH-SY5Y cells, pretreated or not with 10 μg/mL ap, 5 μg/mL mp and 10 μg/mL p *P. lenticonus*/Chios mastiha’s fractions for 24 h and then, subjected or not to 700 μΜ H_2_O_2_ for 6 h. Vehicle-treated, control cells were incubated with 1/1000 *v/v* DMSO. Representative images were taken with a Zeiss LSM-800 confocal microscope in 40× objective. (**B**) Quantification of green to red staining. Data were analyzed by 2-way ANOVA and JC-1 fluorescence relative ratio (JC-1 monomers fluorescence density to relative JC-1 dimers fluorescence density per single cell) is expressed as mean ± SD (*n* = 35–50), * *p* < 0.05, ** *p* < 0.01, *** *p* < 0.001. JC-1 fluorescence ratio of control cells was set as 1.

The protective effect of the *P. lenticonus*/Chios mastiha’s fractions on H_2_O_2_-induced oxidative stress and reduction in cell viability of SH-SY5Y neuroblastoma cells leads us to evaluate the possible protective effect of the *P. lenticonus*/Chios mastiha’s fractions on mitochondrial functionality. Thus, mitochondrial membrane depolarization was assessed in SH-SY5Y cells pretreated or not with *P. lenticonus*/Chios mastiha fractions and subsequently subjected to H_2_O_2_ treatment, using the JC-1 dye. JC-1 monomer/aggregate (green/red) staining was assessed by applying confocal microscopy analysis. As shown in Figure 3, H_2_O_2_ treatment of the cells caused increased depolarization of the mitochondrial membrane as indicated by the increased ratio of the green to red staining compared to control vehicle-treated cells. Incubation of SH-SY5Y cells with the ap, mp, and p fractions of *P. lenticonus*/Chios mastiha, at concentrations of 10 μg/mL, 5 μg/mL, and 10 μg/mL, respectively, did not cause any mitochondrial depolarization, in accordance with results from the MTT assay. On the contrary, green/red JC1 staining was lower in the presence of ap and p fractions compared to control vehicle-treated cells, indicating the potential beneficial effect of the ap and p fractions on the preservation of the mitochondrial membrane potential. Interestingly, preincubation of the H_2_O_2_-treated SH-SY5Y cells with the different polarity fractions of *P. lenticonus*/Chios mastiha led to the maintenance of the mitochondrial membrane potential even in the presence of the oxidizing agent.

### 2.5. Regulation of GR, PPARα και Bcl-2 Protein Levels by the Apolar Fraction

**Figure 4:**
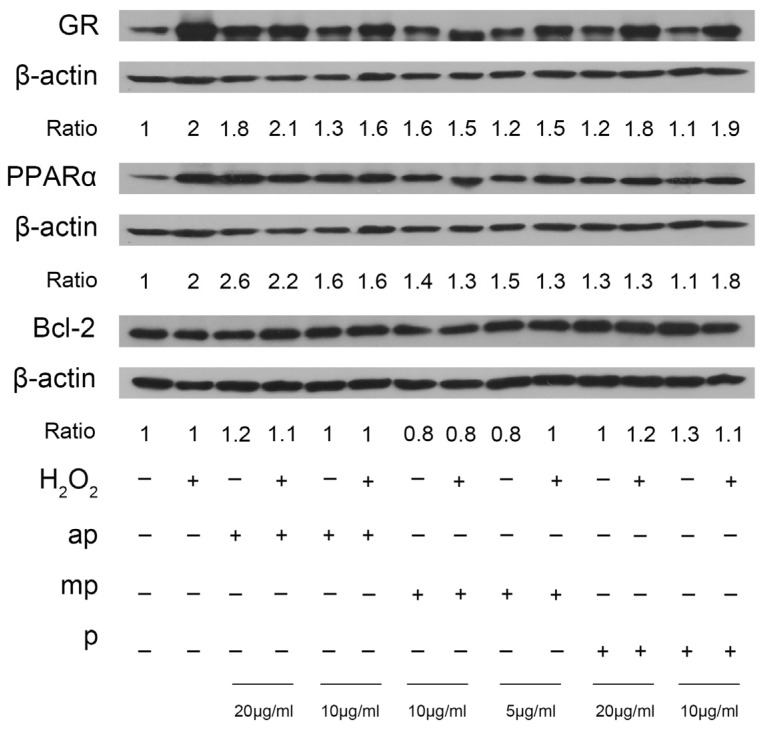
Regulation of the GR, PPARα, and Bcl-2 protein levels by the three different polarity fractions (ap, mp, p) of *P. lenticonus*/Chios mastiha. SH-SY5Y cells were treated with 20 μg/mL and 10 μg/mL ap and p *P. lenticonus*/Chios mastiha’s fractions and 10 μg/mL and 5 μg/mL with mp fraction for 24 h and subsequently treated with 700 μΜ H_2_O_2_ for 6 h. Vehicle cells were incubated with 1/1000 *v/v* DMSO. Western blot analysis results were expressed as the ratios of the GR, PPARα, and Bcl-2 band intensity normalized against the respective band intensity of the β-actin. Relative protein levels in control cells were set to 1.

Taking into consideration the fatty acid detection in *P. lenticonus*/Chios mastiha’s fractions and the crucial role of PPARα, a fatty acid-activating factor, in the regulation of many cellular functions, including lipid metabolism, antioxidant, and anti-inflammatory actions, the effect of *P. lenticonus*/Chios mastiha’s resin fractions on the modulation of PPARα protein levels was assessed (Figure 4). Western blot analysis of PPARα in extracts of SH-SY5Y cells pretreated with the ap, mp, and p *P. lenticonus*/Chios mastiha’s fractions and subsequently subjected to H_2_O_2_ treatment revealed a dose-dependent increase (1.3 to 2.6 folds increase) in PPARα protein levels. The highest activity was observed by the ap fraction. Considerable induction of PPARα levels was also observed with the 700 μΜ H_2_O_2_ treatment. A similar dose-dependent effect, by the ap and mp fractions, was also observed on GR protein levels, whose activity is proposed to be regulated by triterpenes and to constitute an activator of PPARα expression [44,45]. Evaluation of *P. lenticonus*/Chios mastiha fractions on the anti-apoptotic Bcl-2 molecule showed a moderate increase (20%) in the Bcl-2 protein levels by the ap fraction and approximately 20% decrease by the mp fraction.

## 3. Discussion

*P. lenticonus*/Chios mastiha is well-known for its enrichment in terpenoids [37,38,40,41]. In this study, we attempted to assess in depth the chemical composition of *P. lenticonus*/Chios mastiha concerning the presence of other chemical groups, such as fatty acids and unidentified compounds in three different polarity fractions (ap, mp, and p). Thus, HPLC-QTOF-MS/MS analysis was applied. Results from the analysis revealed 22 new components, mainly fatty acid compounds in *P. lenticonus*/Chios mastiha. Enrichment of the apolar and medium polar fraction with fatty acids such as palmitic, stearic, arachidonic, and pentadecanoic acids—compounds known to be involved in cellular signaling, affecting, among others, lipid metabolism and inflammatory responses [46,47,48,49,50]—was detected. To our knowledge, this is the first time that the enrichment of *P. lenticonus*/Chios mastiha in such fatty acids has been uncovered, shedding light on the further identification of the *P. lenticonus*/Chios mastiha chemical composition and the interpretation of the molecular mechanisms of many of the *P. lenticonus*/Chios mastiha’s biological actions, including anti-inflammatory activities and regulation of fatty acid metabolism. Moreover, the presence and enrichment of the p fraction with betulinic acid and euscaphic acid, terpenoids with known antioxidant and anti-inflammatory activities [51,52,53,54], as well as bioactive fatty acids such as linolenic acid, ricinoleic, and crepenynic acid, were revealed, unwrapping the presence of both saturated and non-saturated fatty acids in the p fraction of *P. lenticonus*/Chios mastiha. Furthermore, the presence of coniferaldehyde, an organic compound with many biological activities, such as anti-inflammatory, antioxidant, cytoprotective [55,56,57,58,59,60], as well as neuroprotective via Nrf2 activation [61], was detected in apolar fraction. Interestingly, a variety of unidentified compounds were also detected in *P. lenticonus*/Chios mastiha’s fractions, which remains to be identified. The plethora of identified and non-identified molecules in *P. lenticonus*/Chios mastiha unveiled its unique chemical composition, which is closely related to the plethora of its biological activities.

To further establish a link between *P. lenticonus*/Chios mastiha’s chemical composition and its biological activities, this study focuses on the investigation of the antioxidant activities of *P. lenticonus*/Chios mastiha; the antioxidant and neuroprotective activities of the three different polarity mastiha fractions—ap, mp, and p—were evaluated in SH-SY5Y cells. Emphasis was placed on the elucidation of the effect of *P. lenticonus*/Chios mastiha’s different polarity fractions on mitochondrial origin ROS production, mitochondrial functionality, the modulation of regulatory factors involved in these processes, and the association of the chemical composition of the fractions with their biological activities.

Evaluation of the potential cytoprotective actions of the *P. lenticonus*/Chios mastiha’s fractions on the H_2_O_2_-induced oxidative stress in SH-SY5Y cells revealed that the *P. lenticonus*/Chios mastiha’s ap fraction exerted the highest antioxidant activity, exhibiting statistically significant resistance to H_2_O_2_-induced reduction in cell viability of SH-SY5Y cells, which was accompanied by resistance to H_2_O_2_-induced increase in ROS production and in mitochondrial membrane depolarization. Enrichment of the apolar fraction with triterpenoids may be responsible for its antioxidant activity. The terpenoid-related antioxidant activities of *P. lenticonus*/Chios mastiha are supported by previous observations demonstrating that triterpenes of *P. lenticonus*/Chios mastiha are responsible for the decrease in LDL oxidation [13], reduction in several oxidative stress biomarkers [15], and enhancement of the antioxidant status in NAFLD obese patients [17]. Interestingly, results from the chemical analysis of this study uncovered enrichment of apolar fraction with fatty acids, such as arachidonic, palmitic, stearic acid, and pentadecanoic acid, which constitute signaling molecules with known antioxidant and/or neuroprotective activities [36,49,60,62,63,64,65,66,67]. Thus, the fatty acid components in the ap fraction of the *P. lenticonus*/Chios mastiha may also contribute to its antioxidant activity in SH-SY5Y neuroblastoma cells. In addition, enhanced antioxidant activities of the apolar fraction may be associated with its enrichment of the potential neuroprotective factor coniferaldehyde (55), which is first demonstrated as a *P. lenticonus*/Chios mastiha compound in this study, as well as with the presence of the octyl formate compound, demonstrated as a mitochondrial biogenesis factor [68].

Resistance to H_2_O_2_-induced decreases in SH-SY5Y cell viability, increases in ROS production, and mitochondrial membrane depolarization was also observed by the mp fraction, which was enriched in fatty acids, as shown in this study, and in triterpenes such as lupeol, oleanolic aldehyde, and triterpenoid methyl esters such as 24Z-masticadienonic acid methyl ester and 24Z-isomasticadienonic acid methyl ester, as we have previously shown [12]. The considerable antioxidant activities of the medium polar fraction may be attributed to fatty acids and to lupeol, which has been proposed as an antioxidant and anti-inflammatory agent with a potential impact on oxidative stress resistance in Alzheimer’s disease [34,69], as well as to its enrichment in the methoxycinnamic acid, as revealed by this study. Methoxycinnamic acid is proposed, among others, as a neuroprotective factor [70].

Structural similarities of triterpenoids with glucocorticoids [27] prompted us to evaluate whether the antioxidant activity of *P. lenticonus*/Chios mastiha’s fractions is related to the interference of their components with glucocorticoid signaling. We observed that the ap fraction caused an increase in GR protein levels, which was accompanied by an increase in PPARα that constitutes a GR target [44,45]. This observation shed light on the molecular mechanism of the potent antioxidant activity of ap and mp fractions of *P. lenticonus*/Chios mastiha since PPARα is considered an antioxidant factor, inducing among others catalase expression, and its expression is known to be triggered in the presence of antioxidant factors [71,72,73,74]. Apolar fraction-induced increase in PPARα protein level may be associated with the enrichment of the fraction with palmitic and stearic fatty acids, which are known as PPARα agonists and induce PPARα gene expression [46,75,76]. Arachidonic acid, an ap fraction identified compound, is known to interact with the anti-inflammatory effects of GR signaling [77] and may also contribute to the ap-induced anti-inflammatory actions [12].

Polar fraction of *P. lenticonus*/Chios mastiha also showed antioxidant activity, via reversal of the H_2_O_2_-induced increase in ROS production and mitochondrial membrane depolarization in SH-SY5Y cells. Since polar fraction is enriched in phenolic compounds, its antioxidant activity may be associated with these compounds, well-known for their antioxidant activity [78]. Antioxidant—the neuroprotective actions of polar fraction may also be associated with its enrichment in luteolin glucoside and gamma-linolenic acid compounds, as revealed in this study, compounds that have been reported to exert neuroprotection [79,80].

## 4. Conclusions

To conclude, in this study the characterization of 31 new components, mainly fatty acid compounds, such as palmitic, stearic, arachidonic, and pentadecanoic acids, and compounds such as octyl formate and coniferaldehyde in three different polarity fractions of *P. lenticonus*/Chios mastiha was achieved. Moreover, the presence of 250 non-target compounds was uncovered, highlighting the great abundance of bioactive molecules in *P. lenticonus*/Chios mastiha and strengthening the potential of its pharmaceutical exploitation. Most importantly, the potent neuroprotective activity of the *P. lenticonus*/Chios mastiha, accomplished by the preservation of the mitochondrial functionality and regulation of antioxidant and anti-inflammatory factors expression, upon oxidative stress induction in neuroblastoma cells, was documented. Precisely, the antioxidant activity of the three different polarity mastiha fractions, ap, mp, and polar, was revealed in human neuroblastoma SH-SY5Y cells. Apolar fraction enriched in antioxidant factors such as triterpenes, fatty acids, octyl formate, and coniferaldehyde exhibited the highest antioxidant–neuroprotective activities, as documented by the enhanced suppression of the H_2_O_2_-induced reduction in SH-SY5Y cell viability, the induction of the antioxidant and anti-inflammatory factors PPARα and GR expression, the reduction in ROS production, and the resistance to the depolarization of the mitochondrial membrane potential, in H_2_O_2_-treated neuroblastoma SH-SY5Y cells. Antioxidant activity, although to a lower extent, was also observed by the mp and p fractions, which can be attributed to their enrichment in terpenoids and phenolic compounds, respectively. Moreover, the identification of neuroprotective factors such as methoxycinnamic and leteolin glucoside and gamma-linolenic acid compounds, as components of the mp and p fraction, respectively, was achieved in this study. Their presence may be correlated with the observed antioxidant activity of the mp and p fractions. Taking into account the seriousness of neurodegenerative diseases, the global trend in increasing the number of patients with this health impairment, alongside the world trend in using natural products to improve health or to face mental illness and neurodegeneration [81], the results from the study uncover and highlight the potential application of *P. lenticonus*/Chios mastiha as a neuroprotective agent in neurodegenerative diseases.

## 5. Materials and Methods

### 5.1. Chemicals

Dulbecco’s Modified Eagle Medium (DMEM), Trypsin, Fetal Bovine Serum (FBS), MitoTracker Red CMX Ros (CMX), the 6-carboxy-2′,7′-dichlorodihydrofluorescein diacetate (6-carboxy H2DCFDA), and the (5,5′,6,6′-tetrachloro-1,1′,3,3′-tetraethylbenzimidazolylcarbocyanine iodide) JC-1, were obtained from Thermo Fisher Scientific (GmbH, Basel, Switzerland). The Molecular protein weight marker was purchased from ProteinTech (Rosemont, IL, USA). Cocktail protease inhibitors were purchased from Roche (Mannheim, Germany). Silica gel 60 (70–230 mesh) and Celite^®^ 545 particle size 0.02–0.1 mm, pH 10 (100 g/L, H_2_O, 20 °C), used for vacuum filtration, were purchased from Macherey–Nagel (Düren, Germany). Solvents were from Sigma-Aldrich (Darmstadt, Germany) and were used without any further purification unless stated otherwise. All other chemicals, including H_2_O_2,_ were purchased from Sigma-Aldrich (Darmstadt, Germany).

### 5.2. Chios Mastiha Fractionation and Chemical Characterization

*P. lenticonus*/Chios mastiha was offered by the “Chios Mastiha Growers Association” (https://www.gummastic.gr/en/, accessed on 18 February 2025), which is the certified organization for the production, identification, and commercial exploitation of the *P. lenticonus*/Chios mastiha. *P. lenticonus*/Chios mastiha’s three different polarity fractions were obtained as previously described [12,14]. Details on the methodology applied are presented in the Supplementary Materials (Supplementary Data S1). The methodology applied for the chemical characterization of the three different polarity fractions of *P. lenticonus*/Chios mastiha is followed. Chromatographic analysis was performed on an ExionAC LC system (SCIEX, Framingham, MA, USA) equipped with a controller, two pumps, a degasser, and an auto-sampler. The X500R Q-TOF mass spectrometer (SCIEX, Framingham, MA, USA) was equipped with an electrospray ionization (ESI) turboVTM source and operated in negative ionization mode. TOF-MS and TOF-MS/MS data were acquired using a data-dependent acquisition ESI mode. For the separation of the analytes, a Fortis C18 column (100 mm length, 2.1 mm i.d, 2.6 μm particle size) purchased from Fortis (Cheshire, United Kingdom) was used, thermostated at 40 °C. The mobile phase consisted of: (A) 0.1% (*v/v*) formic acid in water and (B) 0.1% (*v/v*) formic acid in methanol. The flow rate was initially set at 0.2 mL min^−1^, increased to 0.4 mL min^−1^ at 15 min, and decreased to 0.2 mL min^−1^ at 16 min. The elution program was as follows: 0–4 min, 99–61%A; 4–12 min, 61–5% A; 12–15 min, 5% A; 15–16 min, 5–99% A; 16–20 min, 99% A. The QTOF-MS system was equipped with an ESI interface, operating in negative mode with the following settings: spray voltage, 4500 V; heater gas temperature, 55 °C; declustering potential, 80 V. The MS/MS spectra were obtained at a collision energy of 45 eV and a collision energy spread of 15 eV. External calibration was performed before analysis with a cluster solution provided by SCIEX. Additionally, the calibration solution was injected at the beginning of each run for internal calibration and once every five samples during batch acquisition. Mass spectra were recorded in the m/z range from 50 to 1000 at an accumulation time of 0.25 s. MS/MS experiments were conducted in data-dependent acquisition mode at an accumulation time of 0.08 s for the 10 most abundant precursor ions per full scan. Sample acquisition was monitored by SCIEX OS software Version 2.0.1., released in 2019 (© 2019 AB Sciex). Extraction ion chromatograms were generated using SCIEX OS software Version 2.0.1. The non-target screening was carried out using Analytics SCIEX OS software Version 2.0.1. and the SCIEX Natural Products Library.

### 5.3. Cell Culture

SH-SY5Y cells were used in the study. SH-SY5Y is a human neuroblastoma cell line widely used as an in vitro model for a variety of neurobiological analyses [82]. SH-SY5Y cells were obtained from the American Type Culture Collection (ATCC) and were cultured in DMEM 1g/L glucose, with phenol red, 10% *v/v* FBS, 2 mM L-glutamine, and 100 units/mL penicillin/streptomycin at 37 °C and 5% CO_2_. For live cell fluorescence microscopy analysis, cells were maintained in low glucose (1g/L), without phenol red DMEM, supplemented with 2 mM L-glutamine and 100 units/mL penicillin/streptomycin.

### 5.4. Antibodies

For Western blot analysis, specific antibodies against Bcl-2, PPARα, GR, and β-actin were used. Monoclonal antibodies against GR, PPARα, and β-actin were obtained from Santa Cruz Biotechnology (Europe Inc., Heidelberg, Germany) and ProteinTech (Rosemont, IL, USA), respectively. Polyclonal antibodies against Bcl-2 were purchased from Cell Signaling Technology (Leiden, The Netherlands).

### 5.5. Cell Viability Assay-MTT

To investigate the effects of different polarity fractions—ap, mp, and p (based on the gradually increased polarity of the solvents used in the fractionation: petroleum ether solvent for ap fraction, ethyl acetate solvent for mp fraction, and tetrahydrofuran solvent for p fraction)—from *P. lenticonus*/Chios mastiha on the cell viability of SH-SY5Y cells, either in the presence or absence of H_2_O_2_, an MTT assay was applied as previously described [83]. Briefly, 8 × 10^3^ cells were plated in a 96-well plate and cultured in low glucose (1 g/L) DMEM, supplemented with 10% *v/v* FBS, 2 mM L-glutamine, and 100 units/mL penicillin/streptomycin for 24 h. Then, cells were treated with 10 and 20 μg/mL of ap and p *P. lenticonus*/Chios mastiha’s fractions and with 5 and 10 μg/mL of mp fraction (diluted in DMSO) for 24 h. Incubation with 700 μΜ H_2_O_2_ for 6 h followed. Control, vehicle-treated cells were incubated with 1/1000 *v/v* DMSO for 24 h as well. Then, MTT reagent was added at a final concentration of 0.5 μg/mL for 3 h at 37 °C. Finally, the produced formazan crystals were dissolved in 100% isopropanol, upon shaking and the absorbance was measured at 570 nm and 690 nm, as a reference, in a multimode plate reader (EnSpire, PerkinElmer, Beaconsfield, UK). Relative cell viability was expressed as the viability of the cells treated with the indicated concentrations of the respective different polarity *P. lenticonus*/Chios mastiha fractions (ap, mp, and p) and/or H_2_O_2_, compared to the cell viability of the vehicle-untreated (control) cells. The viability of control cells was set to 1.

### 5.6. Electrophoresis and Western Blot Analysis

For Western blot analysis, 25 × 10^4^ SH-SY5Y cells were grown in 6-well plates in low glucose (1g/L glucose) DMEM supplemented with 10% *v/v* FBS, 2 mM L-glutamine, and 100 units/mL penicillin/streptomycin for 48 h. Then, the cells were incubated with 20 μg/mL and 10 μg/mL of ap and p fractions of *P. lenticonus*/Chios mastiha, and with 10 μg/mL and 5 μg/mL of the medium polar fraction, for 24 h. Subsequently, SH-SY5Y cells were further incubated with 700 μΜ H_2_O_2_ for 6 h, or 1/1000 *v/v* DMSO (control vehicle-treated cells). Then, cells were washed with PBS (4 °C) and lysed in lysis buffer (20 mM Tris-HCl pH: 7.5, 250 mM NaCl, 0.5 % *v/v* Triton-X, 3 mM EDTA, supplemented also with DTT, PMSF, and cocktail of protease inhibitors). Bradford assay was applied for protein determination [12]. Then, cell extracts were electrophoresed in discontinuous SDS-PAGE and Western blotting with specific antibodies against Bcl-2, GR, PPARα, and β-actin, as previously described [84]. Enhanced chemiluminescence was used for the detection of the protein bands. Quantification of protein band density was performed by the ImageJ program (v1.52). β-actin band intensity was used for the normalization of the results. Relative protein levels were expressed as band intensity normalized against the respective band’s intensity of β-actin. Relative protein levels in control vehicle-treated cells were set to 1 [29].

### 5.7. Intracellular ROS Measurement

To evaluate the antioxidant activity of different polarity fractions from *P. lenticonus*/Chios mastiha, total ROS production measurements were conducted in SH-SY5Y cells pretreated with the ap, mp, and p fractions and then subjected to H_2_O_2_-induced oxidative stress. The 6-carboxy-2′,7′-dichlorodihydrofluorescein diacetate, (6-carboxy H2DCFDA) dye (Thermo Fisher Scientific) was used for ROS detection. The cell-permeant H2DCFDA is a non-fluorescent indicator for reactive oxygen species (ROS) in cells. Upon cleavage of the acetate groups by intracellular esterases and oxidation, the nonfluorescent H2DCFDA is converted to the highly fluorescent 2′,7′-dichlorofluorescein (DCF). Thus, for ROS production measurement, 1 × 10^5^ SH-SY5Y cells were plated on 60 × 15 mm culture dishes and cultured in low glucose (1 g/L) DMEM, supplemented with 10% *v/v* FBS, 2 mM L-glutamine, and 100 units/mL penicillin/streptomycin for 48 h. Then, cells were incubated with 10 μg/mL of ap, and p fractions or with 5 μg/mL of mp fraction for 24 h. Vehicle (1/1000 *v/v* DMSO)-treated cells were used as control. Subsequently, cells were subjected to H_2_O_2_ (700 μΜ) or vehicle (1/1000 H_2_O) treatment for 6 h. Then, the cell culture medium was replaced with low glucose (1 g/L glucose), without phenol red DMEM, supplemented with 2 mM L-glutamine and 100 units/mL penicillin/streptomycin, containing 10 μΜ H2DCFDA dye, 200 nM CMX and 1 μg/mL Hoecsht-33342 at 37 °C for 30 min. Finally, cells were washed in DMEM without phenol red and subjected to live cell imaging using confocal microscopy analysis. Fluorescence excitation of the oxidized DCF-DA dye was at 488 nm, and fluorescence emission was measured at 530–550 nm using a Zeiss LSM 800 confocal microscope [85].

### 5.8. Analysis of the Mitochondrial Membrane Potential

To assess the ability of *P. lenticonus*/Chios mastiha’s fractions to reverse the H_2_O_2_-induced mitochondrial membrane depolarization of SH-SY5Y cells, mitochondrial membrane potential changes were measured using (5,5′,6,6′-tetrachloro-1,1′,3,3′-tetraethylbenzimidazolylcarbocyanine iodide) JC-1 dye. JC-1 is a lipophilic cationic dye that can enter mitochondria, where it accumulates and forms reversible red fluorescent J-aggregates. In apoptotic cells, the JC-1 dye also enters the mitochondria but to a lesser degree since the inside of the mitochondria is less negative. Under such circumstances, JC-1 does not reach the appropriate concentration to trigger the formation of J aggregates, thus retaining it as a green, fluorescent dye. Based on that, an assessment of the green/red fluorescence ratio depicts the state of the mitochondrial membrane polarization [86]. For the JC-1 assay, 1 × 10^5^ SH-SY5Y cells were plated on 60 × 15 mm culture dishes and cultured in low glucose DMEM (1 g/L glucose), supplemented with 10% *v/v* FBS, 2 mM L-glutamine, and 100 units/mL penicillin/streptomycin for 48 h. Then, cells were treated with 10 μg/mL of ap and p fractions, or with 5 μg/mL of mp fraction for 24 h or with 1/1000 *v/v* DMSO (control cells), and subsequently cultured in the presence or absence of 700 μΜ H_2_O_2_ for 6 h. Finally, cells were incubated with 1 μg/mL Hoechst-33342 and 2 μΜ JC-1, without phenol red-DMEM culture medium, at 37 °C for 30 min. Then, DMEM was replaced with a fresh medium, and cells were subjected to confocal microscopy live imaging analysis. Green JC-1 fluorescence was measured at an excitation of 488 nm, and the emission was measured at 494–535 nm. The red fluorescent J-aggregates were measured at an excitation of 560 nm, and the emission wavelength was at 560–590 nm, using a Zeiss LSM 800 confocal microscope [85].

### 5.9. Statistical Analysis

Results are expressed as mean ± SD. Data were analyzed by one-way analysis of variance (one-way ANOVA) or two-way ANOVA followed by Tukeys’s post hoc test using StatPlus LE Software, version 7.0 (AnalystSoft, Brandon, FL, USA). Differences were considered significant at a two-tailed *p* value < 0.05.

## Figures and Tables

**Table 1:** Suspect compounds by HPLC-QTOF-MS/MS analysis in the *P. lenticonus*/Chios mastiha’s different polarity fractions (ap, mp, and p). Normalized concentrations (μg/L) and their relative enrichment are presented.

Compounds	Quantitative Measurement (μg/L)			Relative Enrichment		
	Apolar, ap	Medium Polar, mp	Polar, p	Apolar, ap	Medium Polar, mp	Polar, p
Coniferaldehyde	103.9	-	-	-	-	-
Palmitic acid	9174.3	1768.3	1102.9	8.3	1.6	1.0
Stearic acid	13,951.4	2437.4	1620.9	8.6	1.5	1.0
Myristic acid	395.7	69.3	79.8	5.7	1.0	1.2
Arachidonic acid	25.9	7.0	0.6	43.2	11.7	1.0
Pentadecanoic acid	246.2	48.9	38.8	6.3	1.3	1.0
Betulinic acid	2119.7	11,462.9	11,725.7	1.0	5.4	5.5
Euscaphic Acid	40.4	199.6	482.0	1.0	4.9	11.9
Flavidin	30.7	265.9	148.1	1.0	8.7	4.8
Luteolin glucoside	0.4	2.2	2.3	1.0	5.5	5.8
α-Linolenic acid	23.0	55.7	103.6	1.0	2.4	4.5
Hesperidin	4.9	6.0	5.3	1.0	1.2	1.1
Oleic acid	2502.3	1443.1	1131.4	2.2	1.3	1.0
Linoleic acid	267.3	248.3	320	1.1	1.0	1.3
Caprylic acid	54.1	35.4	31.7	1.7	1.1	1.0
Nebraskanic acid	47.5	50.0	65.8	1.0	1.1	1.4
Octyl formate	122.2	54.7	40.6	3.0	1.3	1.0
6,7-dihydro-7-hydroxylinalool	64.1	51.3	53.7	1.2	1.0	1.04
α-irone	10.3	18.5	13.5	1.0	1.8	1.3
Oleanonic acid	29,571.4	32,200.0	25,114.3	1.2	1.3	1.0
Resveratrol	5.7	6.4	6.7	1.0	1.1	1.2
Isoliquiritigenin di-glucoside	3.1	0.1	3.1	31.0	1.0	31.0
Methoxycinnamic acid	10.3	18.5	13.5	1.0	1.8	1.3
Masticadecanoic acid	29,571.4	32,200.0	25,114.3	1.2	1.3	1.0
Gamma linolenic acid	23.0	55.7	103.6	1.0	2.4	4.5
Oleanolic acid	601.1	2920.0	2885.7	1.0	4.9	4.8
Ricinoleic acid	23.0	55.7	103.6	1.0	2.4	4.5
Crepenynic acid	23.0	55.7	103.6	1.0	2.4	4.5

**Table 2:** Non-target compounds in the different polarity *P. lenticonus*/Chios mastiha’s fractions as revealed by non-target analysis using the SCIEX Natural Products Library and their chemical classification.

Non-Target Compounds	Non-Target Compounds Chemical Classification
Ethyl 2-acetyl heptanoate	straight-chain fatty acid
Sesterstatin	sesterterpenoid
Cyclohexanecarboxylic acid	organic compound
1,2-Hydroxylauric acid	medium-chain fatty acid
Dodecanedioic acid	saturated aliphatic dicarboxylic acid
Tisocalcitate	organic compound (vitamin D derivative)
Trivalerin	(ester of valeric acid) straight-chain saturated fatty acid
Frangulin B	anthraquinone
4,4′,6,6′-Tetra-tert-butyl-2,2′-biphenol	aromatic hydrocarbon

## Data Availability

All data, tables, and figures are original. Details on data analysis are available from the corresponding author upon reasonable request.

## Supplementary Materials

The following supporting information can be downloaded at: https://www.mdpi.com/article/10.3390/molecules30050997/s1. Supplementary Data S1: Protocol of *P. lenticonus*/Chios mastiha fractionation [87,88,89,90,91,92,93,94,95,96,97,98,99,100,101,102,103,104,105,106,107,108,109,110,111,112,113,114,115,116,117,118,119,120,121,122,123,124,125,126,127,128,129,130,131,132,133,134,135,136,137,138,139,140,141,142,143,144,145,146,147,148,149,150,151,152,153,154,155,156,157,158,159,160,161,162,163,164,165,166,167,168,169,170,171,172,173,174,175,176,177,178,179,180,181,182,183,184,185,186,187,188,189,190,191,192,193,194,195,196,197,198,199,200,201,202,203,204,205,206,207,208,209,210,211,212,213]. Table S1: Suspect compounds identified in different polarity fractions from *P. lenticonus*/Chios mastiha by HPLC-QTOF-MS/MS analysis, their chemical classification, and biological activities. Table S2: Non-target screening results, identified by HPLC-QTOF-MS/MS analysis, in different polarity fractions from mastiha, their chemical classification, and biological activities. Table S3: Unequivocal molecular formulas and hit scores of non-target compounds identified in apolar, medium polar, and polar fractions from *P. lenticonus*/Chios mastiha assigned by SCIEX OS software Version 2.0.1. and the SCIEX Natural Products Library. Figure S1: Extracted Ion Chromatograms (EICs), MS, and MS/MS spectra of suspect compounds; Figure S2: Extracted Ion Chromatograms (EICs), MS, and MS/MS spectra of non-target compounds. References [87,88,89,90,91,92,93,94,95,96,97,98,99,100,101,102,103,104,105,106,107,108,109,110,111,112,113,114,115,116,117,118,119,120,121,122,123,124,125,126,127,128,129,130,131,132,133,134,135,136,137,138,139,140,141,142,143,144,145,146,147,148,149,150,151,152,153,154,155,156,157,158,159,160,161,162,163,164,165,166,167,168,169,170,171,172,173,174,175,176,177,178,179,180,181,182,183,184,185,186,187,188,189,190,191,192,193,194,195,196,197,198,199,200,201,202,203,204,205,206,207,208,209,210,211,212,213] are cited in the Supplementary Materials.
