# Supplementary material for: Chemical Analysis and Antioxidant Activities of Resin Fractions from Pistacia lentiscus L. var. Chia in Neuroblastoma SH-SY5Y Cells
Source: Molecules. 2025 Feb 21;30(5):997. doi: 10.3390/molecules30050997 (PMC11901618; doi:10.3390/molecules30050997)
Supplement: Supplementary file 1 [file molecules-30-00997-s001.zip › molecules-3448788-supplementary.pdf]

Supplementary Materials

Supplementary Data 1. *P. lenticonus*/Chios mastiha fractionation

P. lenticonus/Chios mastiha different polarity fractions (apolar, medium polar and polar) were obtained and analyzed as described by Kalousi et al. [1]. Specifically, 10 g of *P. lenticonus*/Chios mastiha (a batch of the sample is stored in Novara Phytochemical Laboratory with code PLR-Chios) was dissolved in acetone (100 mL volume acetone/plant material weight, 10:1) in a 250 mL round bottom flask upon stirring at room temperature. After 2 h, the dissolved extract was vacuum-filtered in a sintered funnel protected by a paper filter to remove the insoluble residue and the solvent evaporated at reduced pressure to afford 9.5 g (95%) of a yellow, creamy syrup. This latter residue was dissolved in 10 mL of acetone, silica gel was added (28.5 g, 1:3 weight extract/weight silica) and the suspension evaporated at reduced pressure to obtain a pale-yellow powder. Subsequently, the powder was stratified on a layer of Celite, (28.5 g, 1:3 weight extract/weight Celite) previously moistened with petroleum ether (40-60) and protected on its surface by a filter paper in a sintered funnel with side arm for vacuum connection following the method described by Kalousi and colleagues [1]. The sample displayed was vacuum-filtered adding solvents of increasing polarity: petroleum ether (Pe, 285 mL, 1:30 weight extract/ mL solvent), ethyl acetate (EtOAc, 285 mL, 1:30 weight extract/ mL solvent) and tetrahydrofuran (THC, 285 mL, 1:30 weight extract/ mL solvent) to afford, after evaporation of each solvent at reduced pressure, apolar fraction (ap) (Pe, 20.1% yield), medium polar fraction (mp) (EtOAc, 61.2% yield), and polar fraction (p) (THF, 5.9% yield) respectively. Petroleum ether, the most apolar solvent, displays affinity for apolar natural compounds. Ethyl acetate, the medium polarity solvent, exhibits affinity for medium polar compounds, while tetrahydrofuran has the higher interaction with polar compounds.

Suspect compounds	Chemical classification	Biological activities
**Betulinic acid**	Triterpene	Anti-cancer [2-4]<br>Anti-inflammatory [4-6]<br>Anti-diabetic [4, 7, 8]
**Coniferaldehyde**	Phenolic compound	Neuroprotective [9]<br>Anti-apoptotic, anti-inflammatory [10]<br>Anti-diabetic [11]
**Euscaphic acid**	Triterpenoid	Anti-cancer [12]<br>Anti-inflammatory [13, 14]
**Flavidin**	Phenolic compound, phenanthrene	Anti-oxidant [15]<br>Anti-inflammatory [16, 17]
**Isoliquiritigenin di-glucoside**	Flavonoid	Anti-cancer [18, 19]
**Luteolin glucoside**	Flavonoid	Anti-inflammatory [20]<br>Anti-cancer [21]<br>Pro-apoptotic [22]<br>Neuroprotective [23]
**Palmitic acid**	Saturated fatty acid	Anti-cancer [24, 25]<br>Anti-obesity agent [26]<br>Human development [27]

		Metabolism/cardiovascular factor [28-32]<br>PPARα endogenous ligand [33]
**Stearic acid**	Saturated fatty acid	PPARα endogenous ligand [33]<br>Anti-diabetic, anti-inflammatory [34]<br>Glucose metabolism regulator [28, 31, 32]<br>Neuroprotective [35]<br>Anti-cancer [36]
**α-Linolenic acid**	Polyunsaturated fatty acid	Anti-obesity agent [37, 38]<br>Anti-inflammatory, anti-oxidant [39, 40]<br>Anti-cancer [41, 42]
**Ricinoleic acid**	Unsaturated fatty acid	Anti-inflammatory [43]<br>Anti-microbial, anti-cancer [44]
**Crepenynic acid**	Fatty conjugate acid	Anti-fungal, regulator of fatty acid homeostasis [45, 46]
**Gamma linolenic acid**	Polyunsaturated fatty acid	Anti-oxidant, anti-inflammatory [47, 48]<br>Anti-cancer, neuroprotective [49-53]
**Myristic acid**	Saturated fatty acid	Anti-bacterial [54]<br>Anxiolytic [55]<br>Anti-fungal [56]<br>Anti-inflammatory [57, 58]<br>PPARα transactivation regulator [59]
**Arachidonic acid**	Polyunsaturated fatty acid	Growth/development factor [60-62]<br>Neuron function regulator [63-65]<br>Neuroprotective [66]<br>Cell survival regulator [67]<br>Anti-inflammatory [68-71]<br>Cardioprotective [72-74]<br>Anti-cancer [75-78]<br>PPARα regulator [79]
**Pentadecanoic acid**	Saturated fatty acid	PPARα/δ agonist [80]<br>Anti-cancer, regulator of mitochondrial function, anti-inflammatory [80-84]
**Oleanolic acid**	Triterpenoid	Anti-oxidant, anti-cancer, anti-inflammatory, anti-diabetic, anti-microbial [85-91]

**Hesperidin**	Flavonoid	Anti-cancer, anti-oxidant, anti-inflammatory, Cardiovascular protection [92-94]
**Oleic acid**	Fatty acid	Anti-cancer [95, 96] anti-inflammatory [97, 98]
**Linoleic acid**	Polyunsaturated fatty acid	Anti-cancer [98, 99], anti-inflammatory [100]
**Nebraskanic acid**	Fatty acid	Anti-cancer, anti-microbial, [101, 102]
**Octyl formate**	Phenolic compound	Anti-microbial [103, 104], Mitochondrial biogenesis factor [105]
**6,7-dihydro-7-hydroxylinalool**	Terpene	Anti-oxidant, anti-inflammatory, anti-cancer [106-109]
**α-irone**	Monoterpene	Anti-inflammatory, anti-viral, anti-bacterial [110, 111]
**Oleanonic acid**	Triterpenoid	Anti-microbial [112, 113]
**Masticadecanoic acid**	Saturated fatty acid	Anti-diabetic [114], anti-inflammatory [115]
**Resveratrol**	Polyphenolic phytoalexin	Anti-oxidant, anti-inflammatory, anti-diabetic, cardioprotective, and anti-cancer [116-119]
**Methoxycinnamic acid**	Phenylpropanoid	Anti-diabetic, anti-cancer, antimicrobial, hepato-, and neuroprotective regulator [120-122]
**Caprylic acid**	Fatty acid	Anti-microbial, anti-bacterial [123], anti-oxidant [124], anti-cancer [125]

Table S1. Suspect compounds identified in different polarity fractions from *P. lenticonus*/Chios by HPLC-QTOF-MS/MS analysis, their chemical classification and biological activities.

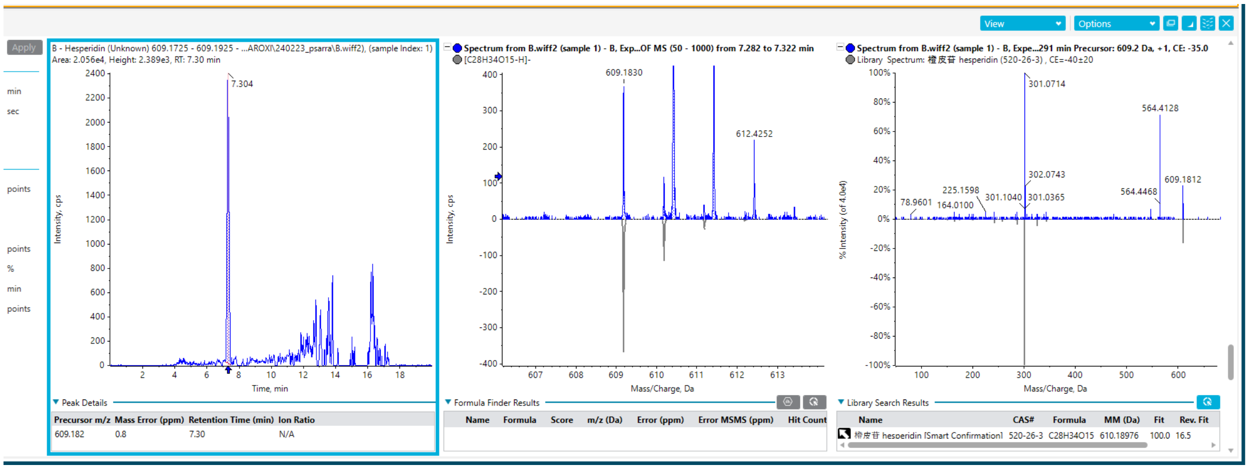
B - Hesperidin (Unknown) 609.1725 - 609.1925 - ...AROX\240223_psarra\B.wiff2), (sample Index: 1)
Area: 2.056e4, Height: 2.389e3, RT: 7.30 min
7.304
Intensity, cps
Time, min
Spectrum from B.wiff2 (sample 1) - B, Exp...OF MS (50 - 1000) from 7.282 to 7.322 min
[C28H34O15-H]-
609.1830
612.4252
Mass/Charge, Da
Spectrum from B.wiff2 (sample 1) - B, Expe...291 min Precursor: 609.2 Da, +1, CE: -35.0
Library Spectrum: 橙皮苷 hesperidin (520-26-3) , CE=-40±20
301.0714
564.4128
302.0743
609.1812
225.1598
564.4468
78.9601
164.0100
301.1040
301.0365
% Intensity (of 4.0e4)
Peak Details
Precursor m/z Mass Error (ppm) Retention Time (min) Ion Ratio
609.182 0.8 7.30 N/A
Formula Finder Results
Name Formula Score m/z (Da) Error (ppm) Error MSMS (ppm) Hit Count
Library Search Results
Name CAS# Formula MM (Da) Fit Rev. Fit
橙皮苷 hesperidin [Smart Confirmation] 520-26-3 C28H34O15 610.18976 100.0 16.5

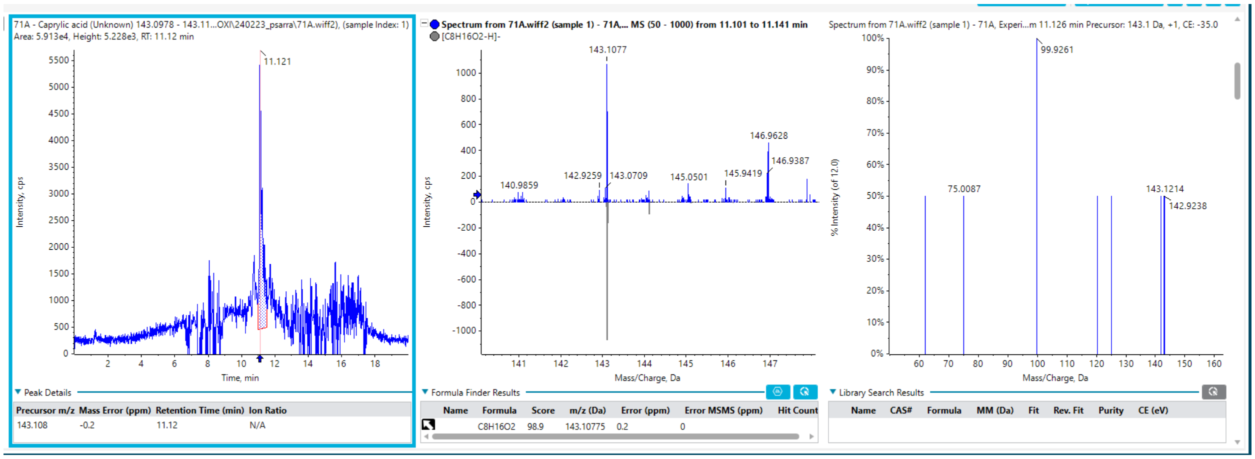
71A - Caprylic acid (Unknown) 143.0978 - 143.11...OX\240223_psarra\71A.wiff2), (sample Index: 1)
Area: 5.913e4, Height: 5.228e3, RT: 11.12 min
11.121
Intensity, cps
Time, min
Spectrum from 71A.wiff2 (sample 1) - 71A,... MS (50 - 1000) from 11.101 to 11.141 min
[C8H16O2-H]-
143.1077
146.9628
146.9387
142.9259
143.0709
145.0501
145.9419
140.9859
Mass/Charge, Da
Spectrum from 71A.wiff2 (sample 1) - 71A, Experi...m 11.126 min Precursor: 143.1 Da, +1, CE: -35.0
99.9261
75.0087
143.1214
142.9238
% Intensity (of 12.0)
Peak Details
Precursor m/z Mass Error (ppm) Retention Time (min) Ion Ratio
143.108 -0.2 11.12 N/A
Formula Finder Results
Name Formula Score m/z (Da) Error (ppm) Error MSMS (ppm) Hit Count
C8H16O2 98.9 143.10775 0.2 0
Library Search Results
Name CAS# Formula MM (Da) Fit Rev. Fit Purity CE (eV)

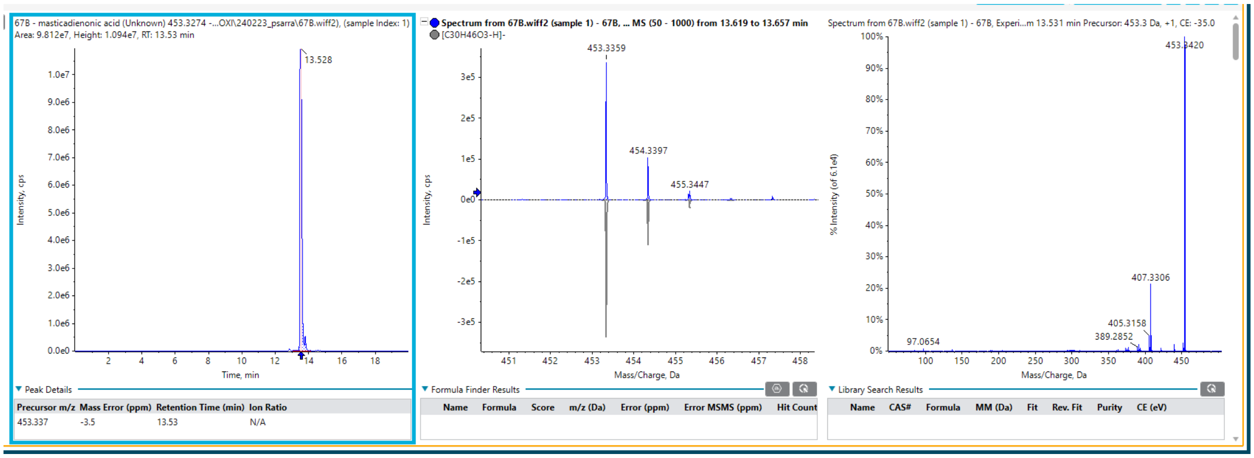
67B - masticadienonic acid (Unknown) 453.3274 - ...OX\240223_psarra\67B.wiff2), (sample Index: 1)
Area: 9.812e7, Height: 1.094e7, RT: 13.53 min
13.528
Intensity, cps
Time, min
Spectrum from 67B.wiff2 (sample 1) - 67B, ... MS (50 - 1000) from 13.619 to 13.657 min
[C30H46O3-H]-
453.3359
454.3397
455.3447
Mass/Charge, Da
Spectrum from 67B.wiff2 (sample 1) - 67B, Experi...m 13.531 min Precursor: 453.3 Da, +1, CE: -35.0
453.3420
407.3306
405.3158
389.2852
97.0654
% Intensity (of 6.1e4)
Peak Details
Precursor m/z Mass Error (ppm) Retention Time (min) Ion Ratio
453.337 -3.5 13.53 N/A
Formula Finder Results
Name Formula Score m/z (Da) Error (ppm) Error MSMS (ppm) Hit Count
Library Search Results
Name CAS# Formula MM (Da) Fit Rev. Fit Purity CE (eV)

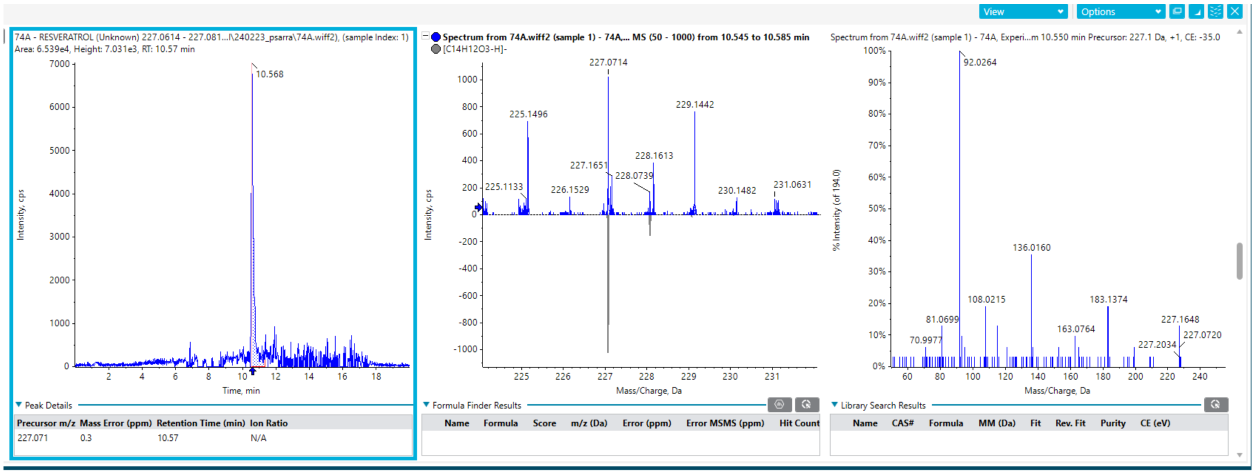
74A - RESVERATROL (Unknown) 227.0614 - 227.081...\240223_psarra\74A.wiff2), (sample Index: 1)
Area: 6.539e4, Height: 7.031e3, RT: 10.57 min
Spectrum from 74A.wiff2 (sample 1) - 74A,... MS (50 - 1000) from 10.545 to 10.585 min
[C14H12O3-H]-
Spectrum from 74A.wiff2 (sample 1) - 74A, Experi...m 10.550 min Precursor: 227.1 Da, +1, CE: -35.0
Intensity, cps
Time, min
Mass/Charge, Da
% Intensity (of 194.0)
Peak Details
Precursor m/z Mass Error (ppm) Retention Time (min) Ion Ratio
227.071 0.3 10.57 N/A
Formula Finder Results
Name Formula Score m/z (Da) Error (ppm) Error MSMS (ppm) Hit Count
Library Search Results
Name CAS# Formula MM (Da) Fit Rev. Fit Purity CE (eV)

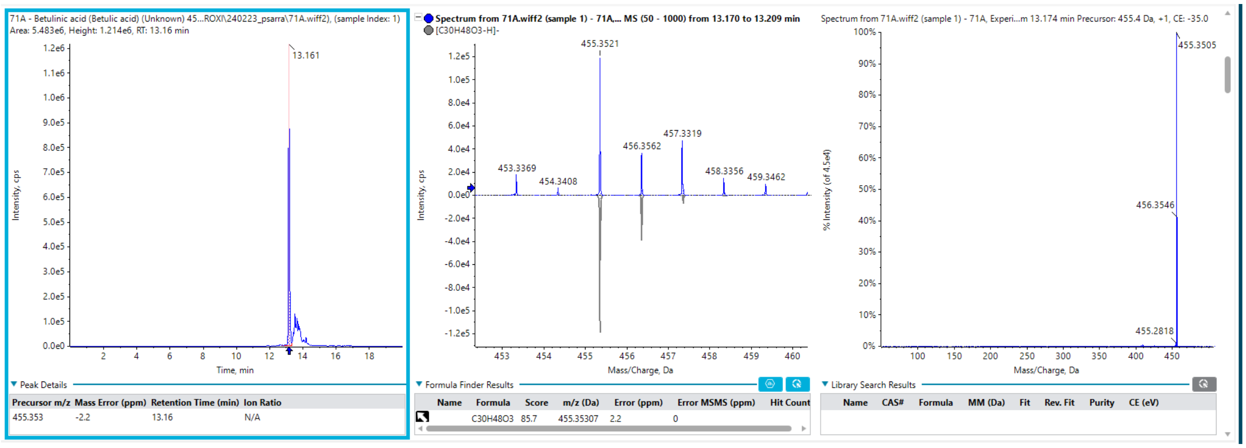
71A - Betulinic acid (Betulic acid) (Unknown) 45...ROX\240223_psarra\71A.wiff2), (sample Index: 1)
Area: 5.483e6, Height: 1.214e6, RT: 13.16 min
Spectrum from 71A.wiff2 (sample 1) - 71A,... MS (50 - 1000) from 13.170 to 13.209 min
[C30H48O3-H]-
Spectrum from 71A.wiff2 (sample 1) - 71A, Experi...m 13.174 min Precursor: 455.4 Da, +1, CE: -35.0
Intensity, cps
Time, min
Mass/Charge, Da
% Intensity (of 4.5e4)
Peak Details
Precursor m/z Mass Error (ppm) Retention Time (min) Ion Ratio
455.353 -2.2 13.16 N/A
Formula Finder Results
Name Formula Score m/z (Da) Error (ppm) Error MSMS (ppm) Hit Count
C30H48O3 85.7 455.35307 2.2 0
Library Search Results
Name CAS# Formula MM (Da) Fit Rev. Fit Purity CE (eV)

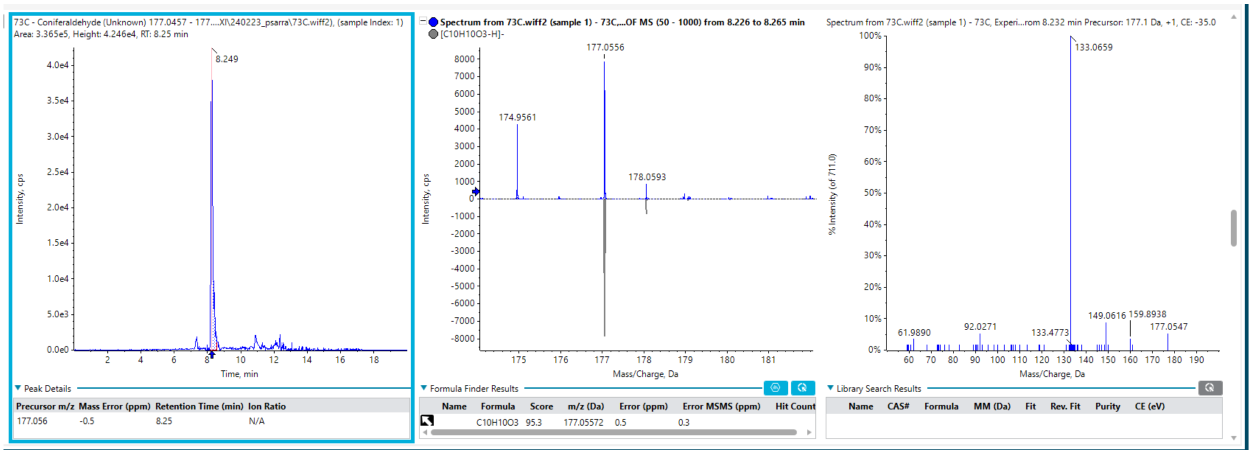
73C - Coniferaldehyde (Unknown) 177.0457 - 177....X\240223_psarra\73C.wiff2), (sample Index: 1)
Area: 3.365e5, Height: 4.246e4, RT: 8.25 min
Spectrum from 73C.wiff2 (sample 1) - 73C,...OF MS (50 - 1000) from 8.226 to 8.265 min
[C10H10O3-H]-
Spectrum from 73C.wiff2 (sample 1) - 73C, Experi...rom 8.232 min Precursor: 177.1 Da, +1, CE: -35.0
Intensity, cps
Time, min
Mass/Charge, Da
% Intensity (of 711.0)
Peak Details
Precursor m/z Mass Error (ppm) Retention Time (min) Ion Ratio
177.056 -0.5 8.25 N/A
Formula Finder Results
Name Formula Score m/z (Da) Error (ppm) Error MSMS (ppm) Hit Count
C10H10O3 95.3 177.05572 0.5 0.3
Library Search Results
Name CAS# Formula MM (Da) Fit Rev. Fit Purity CE (eV)

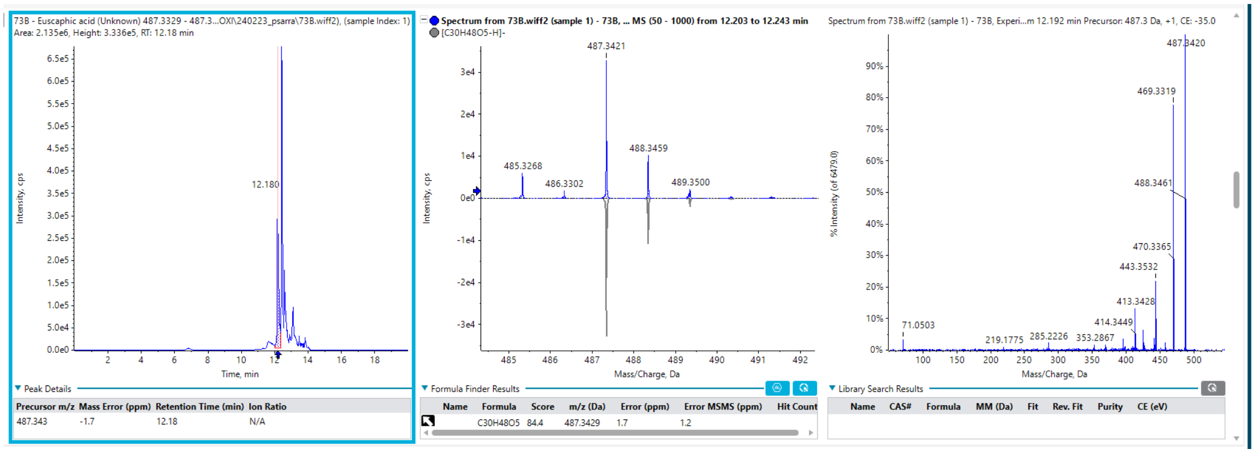

73B - Euscaphic acid (Unknown) 487.3329 - 487.3...OXI\240223_psarra\73B.wiff2), (sample Index: 1)
Area: 2.135e6, Height: 3.336e5, RT: 12.18 min
Spectrum from 73B.wiff2 (sample 1) - 73B, ... MS (50 - 1000) from 12.203 to 12.243 min
[C30H48O5-H]-
Spectrum from 73B.wiff2 (sample 1) - 73B, Experi...m 12.192 min Precursor: 487.3 Da, +1, CE: -35.0
Peak Details
Precursor m/z Mass Error (ppm) Retention Time (min) Ion Ratio
487.343 -1.7 12.18 N/A
Formula Finder Results
Name Formula Score m/z (Da) Error (ppm) Error MSMS (ppm) Hit Count
C30H48O5 84.4 487.3429 1.7 1.2
Library Search Results
Name CAS# Formula MM (Da) Fit Rev. Fit Purity CE (eV)

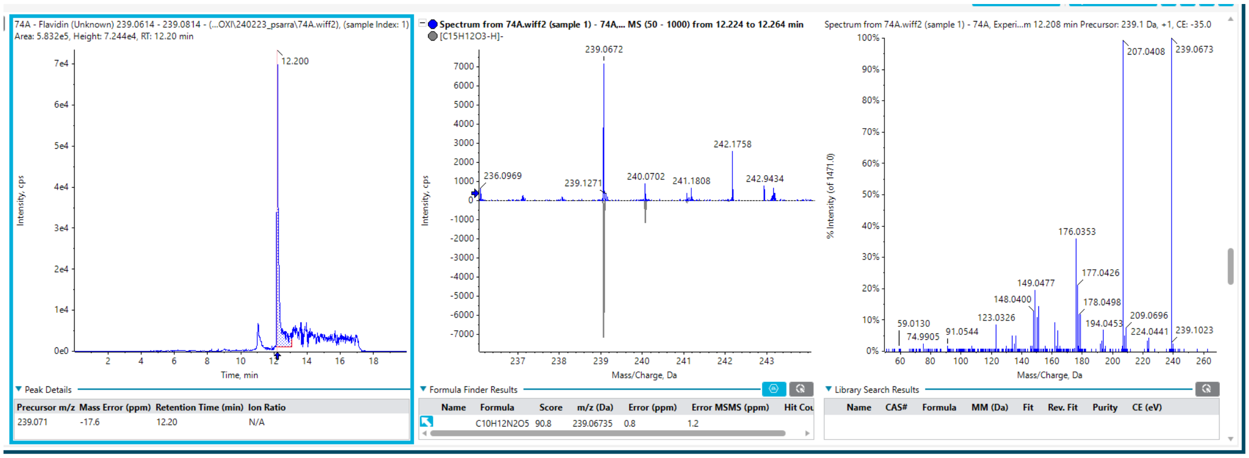

74A - Flavidin (Unknown) 239.0614 - 239.0814 - (...OXI\240223_psarra\74A.wiff2), (sample Index: 1)
Area: 5.832e5, Height: 7.244e4, RT: 12.20 min
Spectrum from 74A.wiff2 (sample 1) - 74A,... MS (50 - 1000) from 12.224 to 12.264 min
[C15H12O3-H]-
Spectrum from 74A.wiff2 (sample 1) - 74A, Experi...m 12.208 min Precursor: 239.1 Da, +1, CE: -35.0
Peak Details
Precursor m/z Mass Error (ppm) Retention Time (min) Ion Ratio
239.071 -17.6 12.20 N/A
Formula Finder Results
Name Formula Score m/z (Da) Error (ppm) Error MSMS (ppm) Hit Cou
C10H12N2O5 90.8 239.06735 0.8 1.2
Library Search Results
Name CAS# Formula MM (Da) Fit Rev. Fit Purity CE (eV)

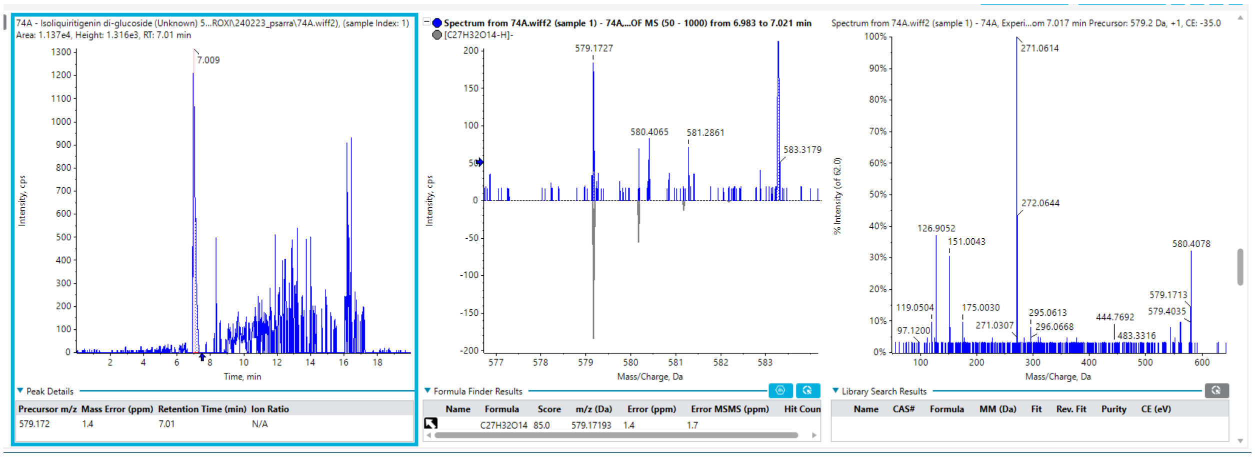

74A - Isoliquiritigenin di-glucoside (Unknown) 5...ROXI\240223_psarra\74A.wiff2), (sample Index: 1)
Area: 1.137e4, Height: 1.316e3, RT: 7.01 min
Spectrum from 74A.wiff2 (sample 1) - 74A,...OF MS (50 - 1000) from 6.983 to 7.021 min
[C27H32O14-H]-
Spectrum from 74A.wiff2 (sample 1) - 74A, Experi...om 7.017 min Precursor: 579.2 Da, +1, CE: -35.0
Peak Details
Precursor m/z Mass Error (ppm) Retention Time (min) Ion Ratio
579.172 1.4 7.01 N/A
Formula Finder Results
Name Formula Score m/z (Da) Error (ppm) Error MSMS (ppm) Hit Coun
C27H32O14 85.0 579.17193 1.4 1.7
Library Search Results
Name CAS# Formula MM (Da) Fit Rev. Fit Purity CE (eV)

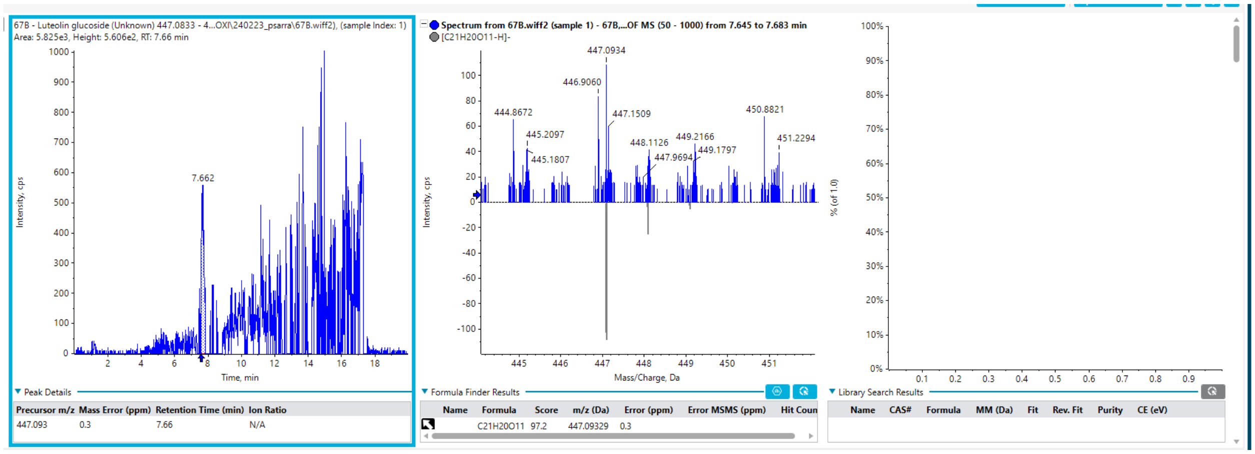
67B - Luteolin glucoside (Unknown) 447.0833 - 4...OXI\240223_psarra\67B.wiff2), (sample Index: 1)
Area: 5.825e3, Height: 5.606e2, RT: 7.66 min
7.662
Intensity, cps
Time, min
Peak Details
Precursor m/z Mass Error (ppm) Retention Time (min) Ion Ratio
447.093 0.3 7.66 N/A
Spectrum from 67B.wiff2 (sample 1) - 67B,...OF MS (50 - 1000) from 7.645 to 7.683 min
[C21H20O11-H]-
447.0934
446.9060
444.8672
447.1509
450.8821
445.2097
448.1126
449.2166
451.2294
445.1807
447.9694
449.1797
Mass/Charge, Da
Formula Finder Results
Name Formula Score m/z (Da) Error (ppm) Error MSMS (ppm) Hit Count
C21H20O11 97.2 447.09329 0.3
% (of 1.0)
Library Search Results
Name CAS# Formula MM (Da) Fit Rev. Fit Purity CE (eV)

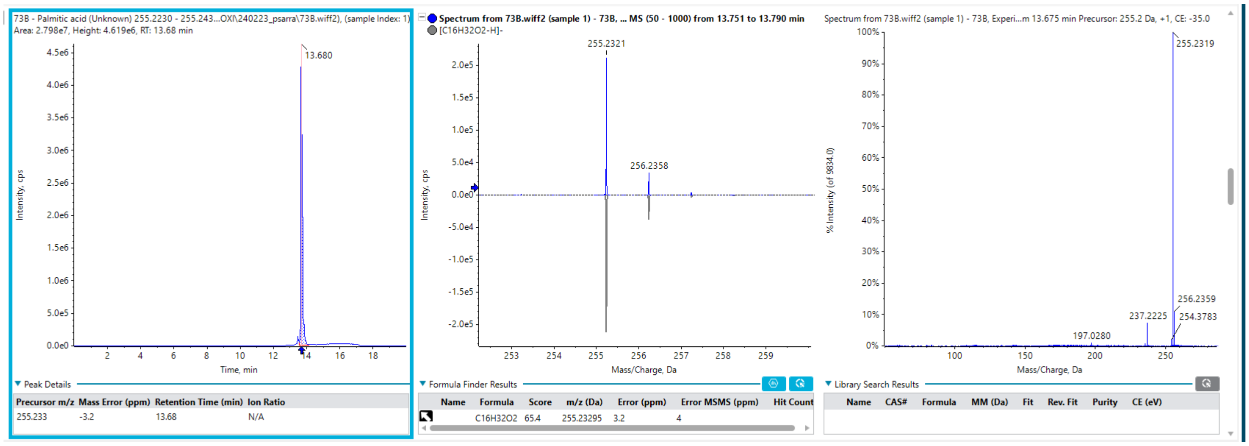
73B - Palmitic acid (Unknown) 255.2230 - 255.243...OXI\240223_psarra\73B.wiff2), (sample Index: 1)
Area: 2.798e7, Height: 4.619e6, RT: 13.68 min
13.680
Intensity, cps
Time, min
Peak Details
Precursor m/z Mass Error (ppm) Retention Time (min) Ion Ratio
255.233 -3.2 13.68 N/A
Spectrum from 73B.wiff2 (sample 1) - 73B, ... MS (50 - 1000) from 13.751 to 13.790 min
[C16H32O2-H]-
255.2321
256.2358
Mass/Charge, Da
Formula Finder Results
Name Formula Score m/z (Da) Error (ppm) Error MSMS (ppm) Hit Count
C16H32O2 65.4 255.23295 3.2 4
Spectrum from 73B.wiff2 (sample 1) - 73B, Experi...m 13.675 min Precursor: 255.2 Da, +1, CE: -35.0
% Intensity (of 9834.0)
255.2319
256.2359
237.2225
254.3783
197.0280
Mass/Charge, Da
Library Search Results
Name CAS# Formula MM (Da) Fit Rev. Fit Purity CE (eV)

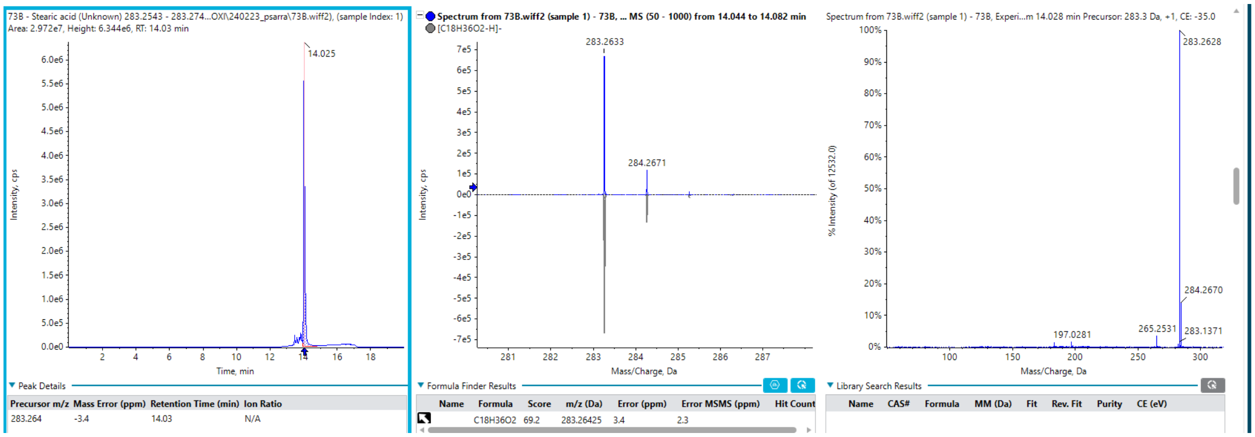
73B - Stearic acid (Unknown) 283.2543 - 283.274...OXI\240223_psarra\73B.wiff2), (sample Index: 1)
Area: 2.972e7, Height: 6.344e6, RT: 14.03 min
14.025
Intensity, cps
Time, min
Peak Details
Precursor m/z Mass Error (ppm) Retention Time (min) Ion Ratio
283.264 -3.4 14.03 N/A
Spectrum from 73B.wiff2 (sample 1) - 73B, ... MS (50 - 1000) from 14.044 to 14.082 min
[C18H36O2-H]-
283.2633
284.2671
Mass/Charge, Da
Formula Finder Results
Name Formula Score m/z (Da) Error (ppm) Error MSMS (ppm) Hit Count
C18H36O2 69.2 283.26425 3.4 2.3
Spectrum from 73B.wiff2 (sample 1) - 73B, Experi...m 14.028 min Precursor: 283.3 Da, +1, CE: -35.0
% Intensity (of 12532.0)
283.2628
284.2670
197.0281
265.2531
283.1371
Mass/Charge, Da
Library Search Results
Name CAS# Formula MM (Da) Fit Rev. Fit Purity CE (eV)

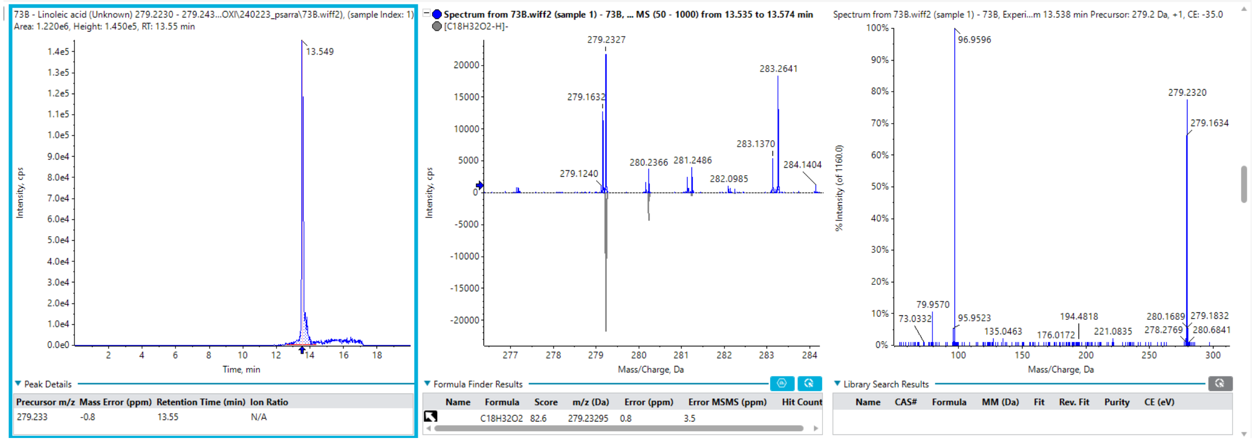
73B - Linoleic acid (Unknown) 279.2230 - 279.243...OXI\240223_psarra\73B.wiff2), (sample Index: 1)
Area: 1.220e6, Height: 1.450e5, RT: 13.55 min
13.549
Intensity, cps
Time, min
Peak Details
Precursor m/z | Mass Error (ppm) | Retention Time (min) | Ion Ratio
279.233 | -0.8 | 13.55 | N/A
Spectrum from 73B.wiff2 (sample 1) - 73B, ... MS (50 - 1000) from 13.535 to 13.574 min
[C18H32O2-H]-
279.2327
283.2641
279.1632
283.1370
280.2366
281.2486
279.1240
284.1404
282.0985
Mass/Charge, Da
Formula Finder Results
Name | Formula | Score | m/z (Da) | Error (ppm) | Error MSMS (ppm) | Hit Count
C18H32O2 | 82.6 | 279.23295 | 0.8 | 3.5
Spectrum from 73B.wiff2 (sample 1) - 73B, Experi...m 13.538 min Precursor: 279.2 Da, +1, CE: -35.0
96.9596
279.2320
279.1634
79.9570
73.0332
95.9523
135.0463
176.0172
194.4818
221.0835
280.1689
278.2769
279.1832
280.6841
% Intensity (of 1160.0)
Mass/Charge, Da
Library Search Results
Name | CAS# | Formula | MM (Da) | Fit | Rev. Fit | Purity | CE (eV)

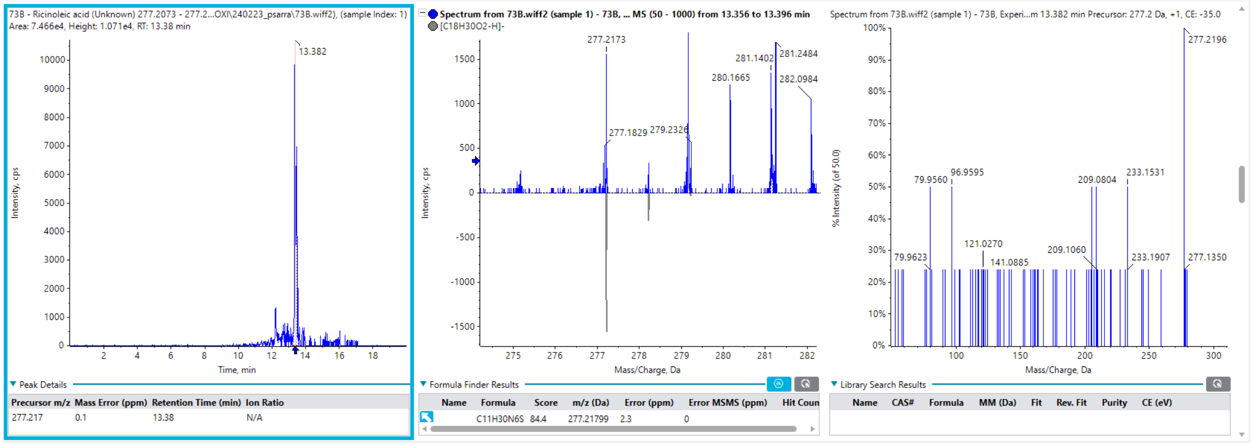
73B - Ricinoleic acid (Unknown) 277.2073 - 277.2...OXI\240223_psarra\73B.wiff2), (sample Index: 1)
Area: 7.466e4, Height: 1.071e4, RT: 13.38 min
13.382
Intensity, cps
Time, min
Peak Details
Precursor m/z | Mass Error (ppm) | Retention Time (min) | Ion Ratio
277.217 | 0.1 | 13.38 | N/A
Spectrum from 73B.wiff2 (sample 1) - 73B, ... MS (50 - 1000) from 13.356 to 13.396 min
[C18H30O2-H]-
277.2173
281.1402
281.2484
280.1665
282.0984
277.1829
279.2326
Mass/Charge, Da
Formula Finder Results
Name | Formula | Score | m/z (Da) | Error (ppm) | Error MSMS (ppm) | Hit Coun
C11H30N6S | 84.4 | 277.21799 | 2.3 | 0
Spectrum from 73B.wiff2 (sample 1) - 73B, Experi...m 13.382 min Precursor: 277.2 Da, +1, CE: -35.0
277.2196
79.9560
96.9595
209.0804
233.1531
121.0270
141.0885
209.1060
79.9623
233.1907
277.1350
% Intensity (of 50.0)
Mass/Charge, Da
Library Search Results
Name | CAS# | Formula | MM (Da) | Fit | Rev. Fit | Purity | CE (eV)

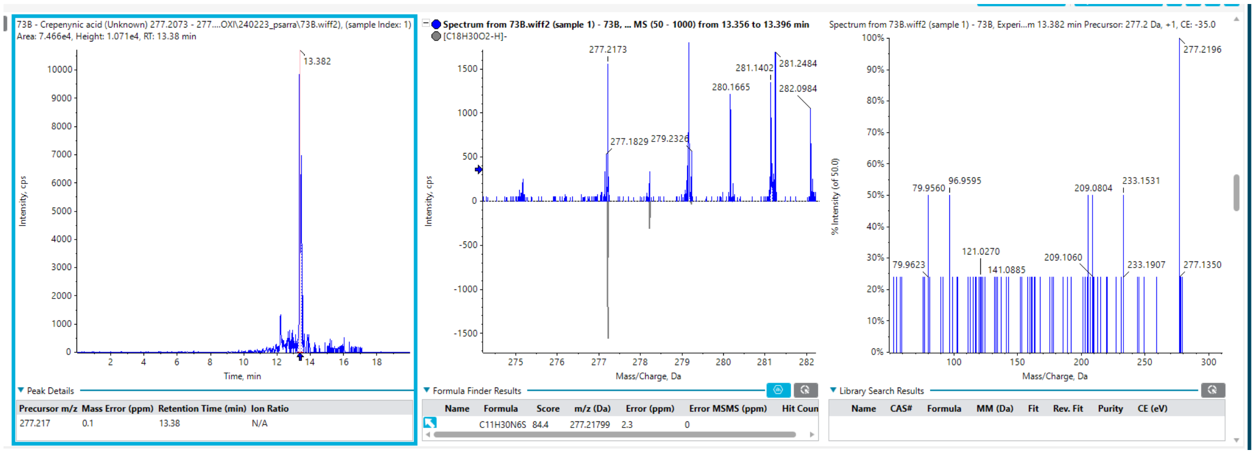
73B - Crepenynic acid (Unknown) 277.2073 - 277....OXI\240223_psarra\73B.wiff2), (sample Index: 1)
Area: 7.466e4, Height: 1.071e4, RT: 13.38 min
13.382
Intensity, cps
Time, min
Peak Details
Precursor m/z | Mass Error (ppm) | Retention Time (min) | Ion Ratio
277.217 | 0.1 | 13.38 | N/A
Spectrum from 73B.wiff2 (sample 1) - 73B, ... MS (50 - 1000) from 13.356 to 13.396 min
[C18H30O2-H]-
277.2173
281.1402
281.2484
280.1665
282.0984
277.1829
279.2326
Mass/Charge, Da
Formula Finder Results
Name | Formula | Score | m/z (Da) | Error (ppm) | Error MSMS (ppm) | Hit Coun
C11H30N6S | 84.4 | 277.21799 | 2.3 | 0
Spectrum from 73B.wiff2 (sample 1) - 73B, Experi...m 13.382 min Precursor: 277.2 Da, +1, CE: -35.0
277.2196
79.9560
96.9595
209.0804
233.1531
121.0270
141.0885
209.1060
79.9623
233.1907
277.1350
% Intensity (of 50.0)
Mass/Charge, Da
Library Search Results
Name | CAS# | Formula | MM (Da) | Fit | Rev. Fit | Purity | CE (eV)

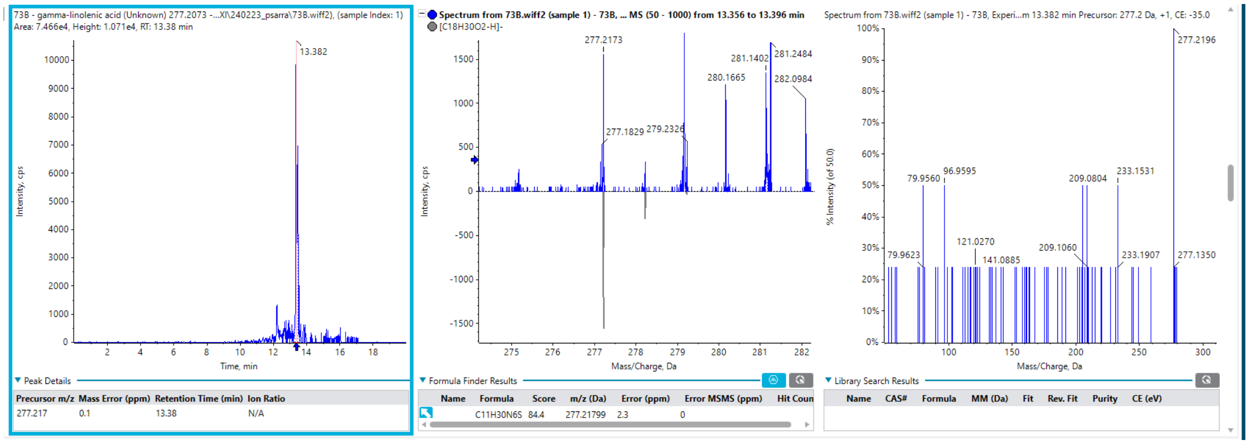
73B - gamma-linolenic acid (Unknown) 277.2073 -...XI\240223_psarra\73B.wiff2), (sample Index: 1)
Area: 7.466e4, Height: 1.071e4, RT: 13.38 min
Spectrum from 73B.wiff2 (sample 1) - 73B, ... MS (50 - 1000) from 13.356 to 13.396 min
[C18H30O2-H]-
Spectrum from 73B.wiff2 (sample 1) - 73B, Experi...m 13.382 min Precursor: 277.2 Da, +1, CE: -35.0
Intensity, cps
Time, min
Mass/Charge, Da
% Intensity (of 50.0)
Peak Details
Precursor m/z | Mass Error (ppm) | Retention Time (min) | Ion Ratio
277.217 | 0.1 | 13.38 | N/A
Formula Finder Results
Name | Formula | Score | m/z (Da) | Error (ppm) | Error MSMS (ppm) | Hit Coun
C11H30N6S | 84.4 | 277.21799 | 2.3 | 0
Library Search Results
Name | CAS# | Formula | MM (Da) | Fit | Rev. Fit | Purity | CE (eV)

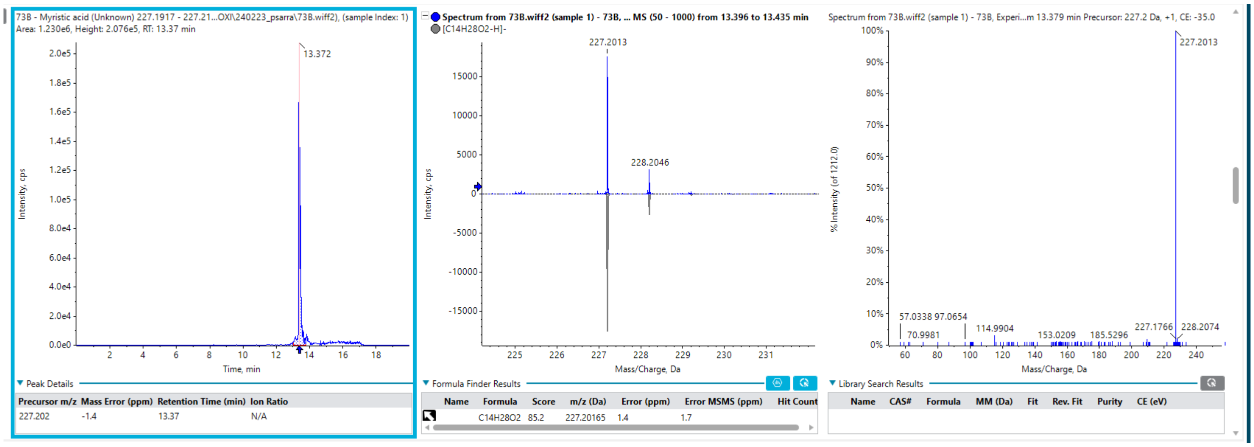
73B - Myristic acid (Unknown) 227.1917 - 227.21...OXI\240223_psarra\73B.wiff2), (sample Index: 1)
Area: 1.230e6, Height: 2.076e5, RT: 13.37 min
Spectrum from 73B.wiff2 (sample 1) - 73B, ... MS (50 - 1000) from 13.396 to 13.435 min
[C14H28O2-H]-
Spectrum from 73B.wiff2 (sample 1) - 73B, Experi...m 13.379 min Precursor: 227.2 Da, +1, CE: -35.0
Intensity, cps
Time, min
Mass/Charge, Da
% Intensity (of 1212.0)
Peak Details
Precursor m/z | Mass Error (ppm) | Retention Time (min) | Ion Ratio
227.202 | -1.4 | 13.37 | N/A
Formula Finder Results
Name | Formula | Score | m/z (Da) | Error (ppm) | Error MSMS (ppm) | Hit Count
C14H28O2 | 85.2 | 227.20165 | 1.4 | 1.7
Library Search Results
Name | CAS# | Formula | MM (Da) | Fit | Rev. Fit | Purity | CE (eV)

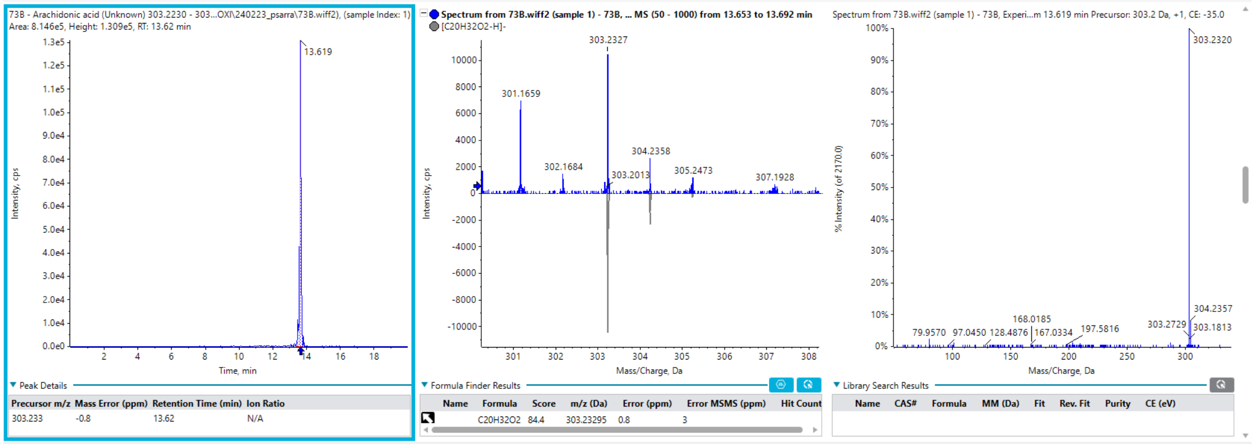
73B - Arachidonic acid (Unknown) 303.2230 - 303...OXI\240223_psarra\73B.wiff2), (sample Index: 1)
Area: 8.146e5, Height: 1.309e5, RT: 13.62 min
Spectrum from 73B.wiff2 (sample 1) - 73B, ... MS (50 - 1000) from 13.653 to 13.692 min
[C20H32O2-H]-
Spectrum from 73B.wiff2 (sample 1) - 73B, Experi...m 13.619 min Precursor: 303.2 Da, +1, CE: -35.0
Intensity, cps
Time, min
Mass/Charge, Da
% Intensity (of 2170.0)
Peak Details
Precursor m/z | Mass Error (ppm) | Retention Time (min) | Ion Ratio
303.233 | -0.8 | 13.62 | N/A
Formula Finder Results
Name | Formula | Score | m/z (Da) | Error (ppm) | Error MSMS (ppm) | Hit Count
C20H32O2 | 84.4 | 303.23295 | 0.8 | 3
Library Search Results
Name | CAS# | Formula | MM (Da) | Fit | Rev. Fit | Purity | CE (eV)

73B - Pentadecanoic acid (Unknown) 241.2073 - 2...XI\240223_psarra\73B.wiff2), (sample Index: 1)
Area: 1.220e6, Height: 1.888e5, RT: 13.52 min

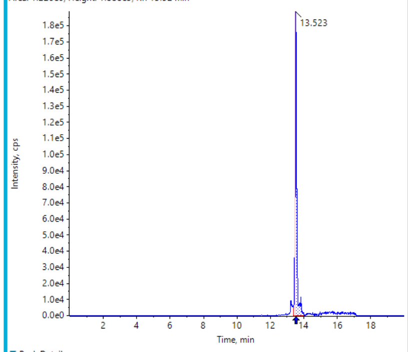

Spectrum from 73B.wiff2 (sample 1) - 73B, ... MS (50 - 1000) from 13.496 to 13.535 min
[C15H30O2-H]-

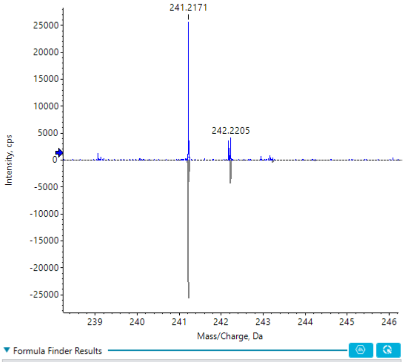

Spectrum from 73B.wiff2 (sample 1) - 73B, Experi...m 13.463 min Precursor: 241.2 Da, +1, CE: -35.0

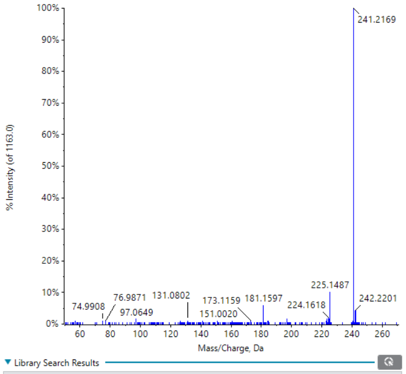

Peak Details

Precursor m/z	Mass Error (ppm)	Retention Time (min)	Ion Ratio
241.217	-0.8	13.52	N/A

Formula Finder Results

Name	Formula	Score	m/z (Da)	Error (ppm)	Error MSMS (ppm)	Hit Count
	C15H30O2	90.2	241.2173	0.8	1.4	

Library Search Results

Name	CAS#	Formula	MM (Da)	Fit	Rev. Fit	Purity	CE (eV)

73C - Oleic acid (Unknown) 281.2386 - 281.2586 ...OXI\240223_psarra\73C.wiff2), (sample Index: 1)
Area: 4.281e6, Height: 7.999e5, RT: 13.75 min

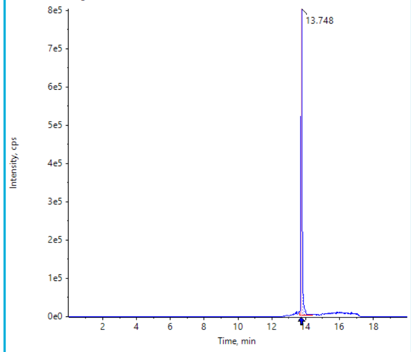

Spectrum from 73C.wiff2 (sample 1) - 73C, ... MS (50 - 1000) from 13.705 to 13.744 min
[C18H34O2-H]-

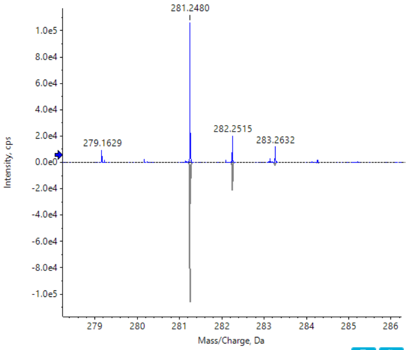

Spectrum from 73C.wiff2 (sample 1) - 73C, Experi...m 13.747 min Precursor: 281.2 Da, +1, CE: -35.0

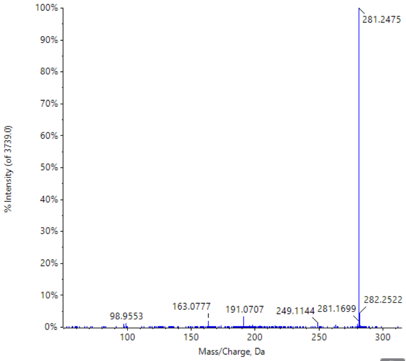

Peak Details

Precursor m/z	Mass Error (ppm)	Retention Time (min)	Ion Ratio
281.249	-2.3	13.75	N/A

Formula Finder Results

Name	Formula	Score	m/z (Da)	Error (ppm)	Error MSMS (ppm)	Hit Count
	C18H34O2	72.1	281.2486	2.3	3.8	

Library Search Results

Name	CAS#	Formula	MM (Da)	Fit	Rev. Fit	Purity	CE (eV)

73C - Linoleic acid (Unknown) 279.2230 - 279.243...OXI\240223_psarra\73C.wiff2), (sample Index: 1)
Area: 1.086e6, Height: 1.843e5, RT: 13.57 min

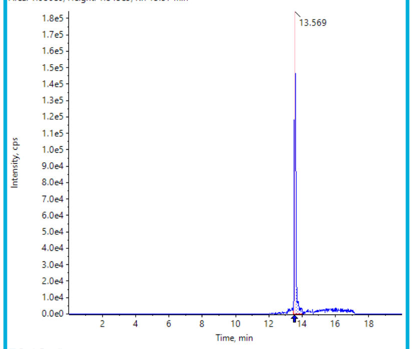

Spectrum from 73C.wiff2 (sample 1) - 73C, ... MS (50 - 1000) from 13.627 to 13.666 min
[C18H32O2-H]-

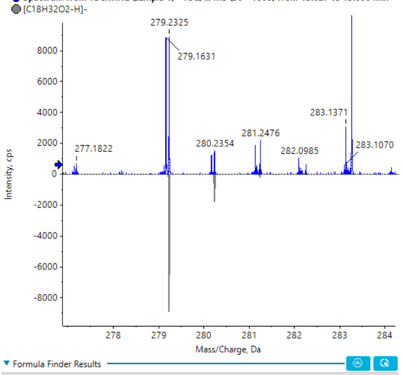

Spectrum from 73C.wiff2 (sample 1) - 73C, Experi...m 13.572 min Precursor: 279.2 Da, +1, CE: -35.0

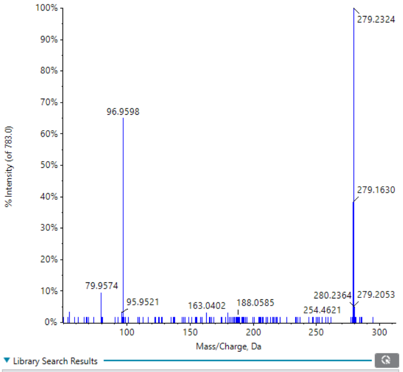

Peak Details

Precursor m/z	Mass Error (ppm)	Retention Time (min)	Ion Ratio
279.233	-1.5	13.57	N/A

Formula Finder Results

Name	Formula	Score	m/z (Da)	Error (ppm)	Error MSMS (ppm)	Hit Count
	C18H32O2	83.8	279.23295	1.5	2	

Library Search Results

Name	CAS#	Formula	MM (Da)	Fit	Rev. Fit	Purity	CE (eV)

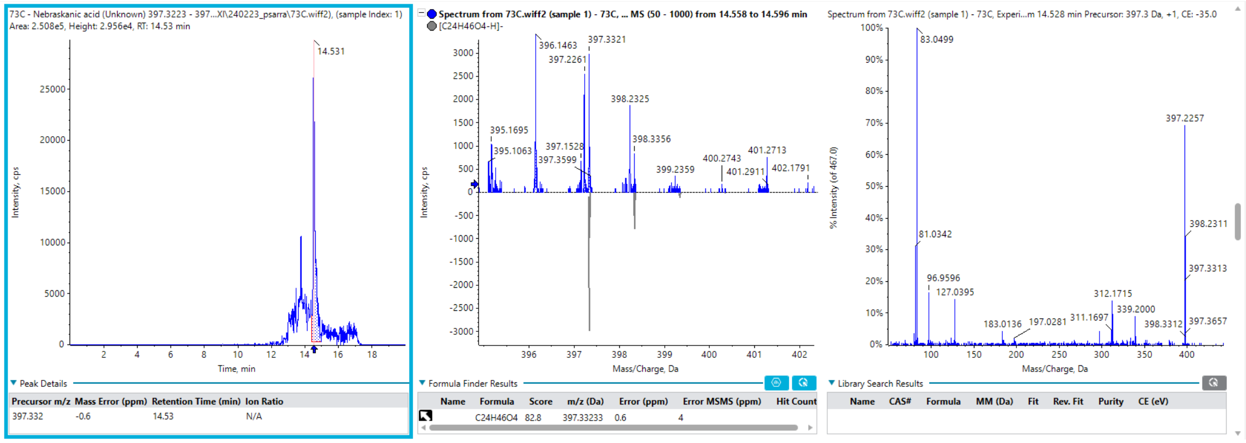
73C - Nebraskanic acid (Unknown) 397.3223 - 397...XI\240223_psarra\73C.wiff2), (sample Index: 1)
Area: 2.508e5, Height: 2.956e4, RT: 14.53 min
14.531
Intensity, cps
Time, min
Peak Details
Precursor m/z | Mass Error (ppm) | Retention Time (min) | Ion Ratio
397.332 | -0.6 | 14.53 | N/A
Spectrum from 73C.wiff2 (sample 1) - 73C, ... MS (50 - 1000) from 14.558 to 14.596 min
[C24H46O4-H]-
396.1463
397.3321
397.2261
398.2325
395.1695
395.1063
397.1528
397.3599
398.3356
400.2743
401.2713
399.2359
401.2911
402.1791
Intensity, cps
Mass/Charge, Da
Formula Finder Results
Name | Formula | Score | m/z (Da) | Error (ppm) | Error MSMS (ppm) | Hit Count
C24H46O4 | 82.8 | 397.33233 | 0.6 | 4
Spectrum from 73C.wiff2 (sample 1) - 73C, Experi...m 14.528 min Precursor: 397.3 Da, +1, CE: -35.0
83.0499
397.2257
81.0342
398.2311
397.3313
96.9596
127.0395
312.1715
339.2000
183.0136
197.0281
311.1697
398.3312
397.3657
% Intensity (of 467.0)
Mass/Charge, Da
Library Search Results
Name | CAS# | Formula | MM (Da) | Fit | Rev. Fit | Purity | CE (eV)

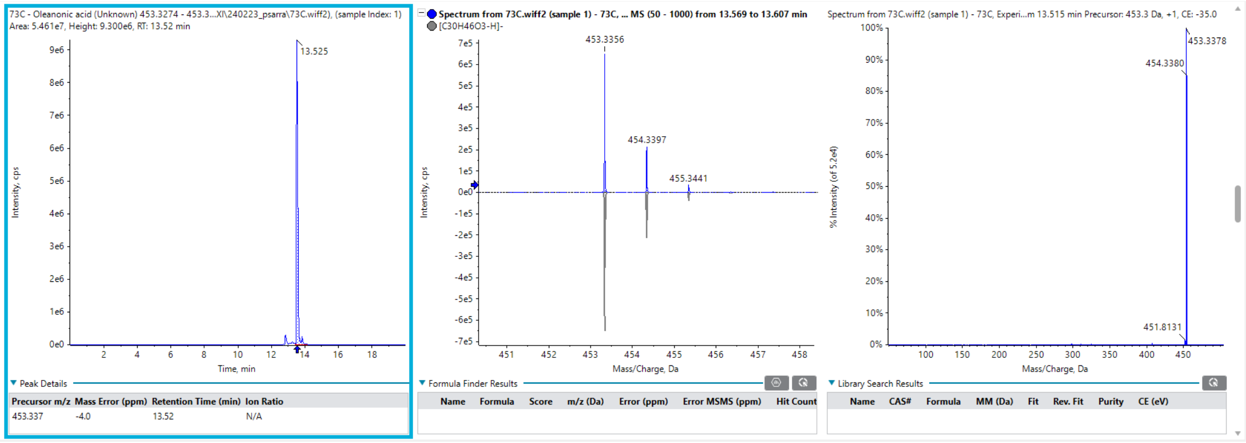
73C - Oleanonic acid (Unknown) 453.3274 - 453.3...XI\240223_psarra\73C.wiff2), (sample Index: 1)
Area: 5.461e7, Height: 9.300e6, RT: 13.52 min
13.525
Intensity, cps
Time, min
Peak Details
Precursor m/z | Mass Error (ppm) | Retention Time (min) | Ion Ratio
453.337 | -4.0 | 13.52 | N/A
Spectrum from 73C.wiff2 (sample 1) - 73C, ... MS (50 - 1000) from 13.569 to 13.607 min
[C30H46O3-H]-
453.3356
454.3397
455.3441
Intensity, cps
Mass/Charge, Da
Formula Finder Results
Name | Formula | Score | m/z (Da) | Error (ppm) | Error MSMS (ppm) | Hit Count
Spectrum from 73C.wiff2 (sample 1) - 73C, Experi...m 13.515 min Precursor: 453.3 Da, +1, CE: -35.0
453.3378
454.3380
451.8131
% Intensity (of 5.2e4)
Mass/Charge, Da
Library Search Results
Name | CAS# | Formula | MM (Da) | Fit | Rev. Fit | Purity | CE (eV)

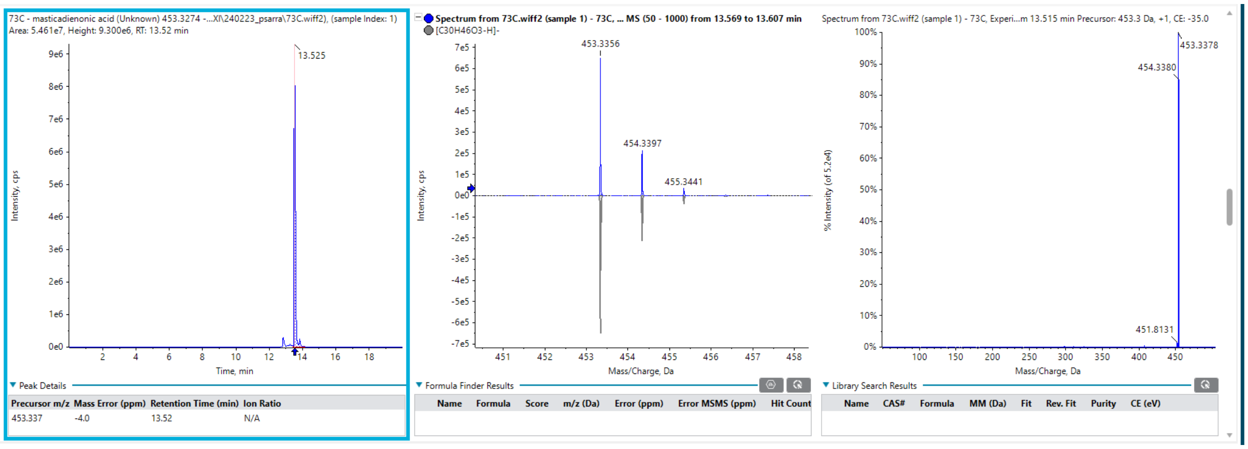
73C - masticadienonic acid (Unknown) 453.3274 -...XI\240223_psarra\73C.wiff2), (sample Index: 1)
Area: 5.461e7, Height: 9.300e6, RT: 13.52 min
13.525
Intensity, cps
Time, min
Peak Details
Precursor m/z | Mass Error (ppm) | Retention Time (min) | Ion Ratio
453.337 | -4.0 | 13.52 | N/A
Spectrum from 73C.wiff2 (sample 1) - 73C, ... MS (50 - 1000) from 13.569 to 13.607 min
[C30H46O3-H]-
453.3356
454.3397
455.3441
Intensity, cps
Mass/Charge, Da
Formula Finder Results
Name | Formula | Score | m/z (Da) | Error (ppm) | Error MSMS (ppm) | Hit Count
Spectrum from 73C.wiff2 (sample 1) - 73C, Experi...m 13.515 min Precursor: 453.3 Da, +1, CE: -35.0
453.3378
454.3380
451.8131
% Intensity (of 5.2e4)
Mass/Charge, Da
Library Search Results
Name | CAS# | Formula | MM (Da) | Fit | Rev. Fit | Purity | CE (eV)

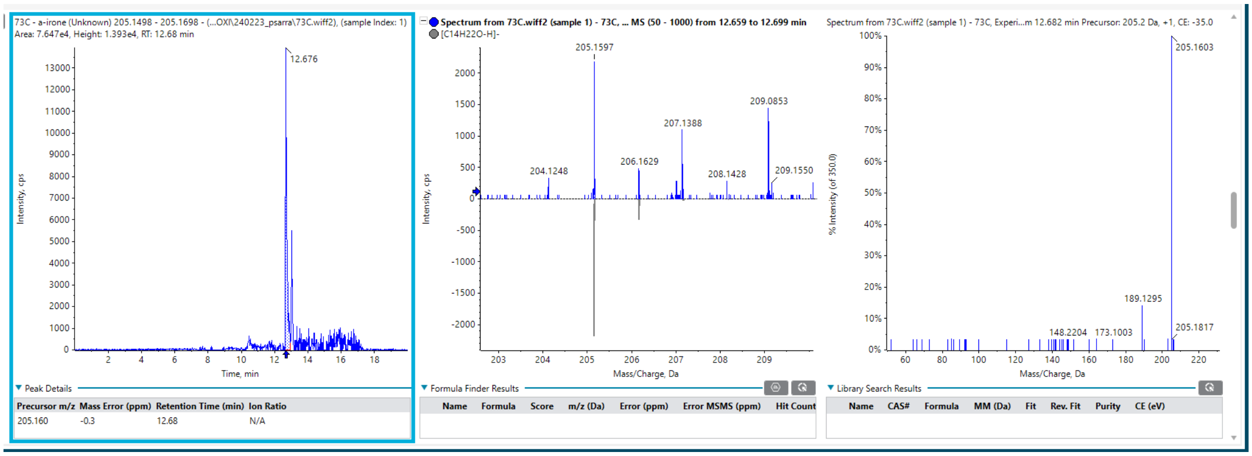
73C - a-irone (Unknown) 205.1498 - 205.1698 - (...OXI\240223_psarra\73C.wiff2), (sample Index: 1)
Area: 7.647e4, Height: 1.393e4, RT: 12.68 min
12.676
Intensity, cps
Time, min
Spectrum from 73C.wiff2 (sample 1) - 73C, ... MS (50 - 1000) from 12.659 to 12.699 min
[C14H22O-H]-
205.1597
209.0853
207.1388
206.1629
204.1248
208.1428
209.1550
Mass/Charge, Da
Spectrum from 73C.wiff2 (sample 1) - 73C, Experi...m 12.682 min Precursor: 205.2 Da, +1, CE: -35.0
205.1603
189.1295
148.2204
173.1003
205.1817
% Intensity (of 350.0)
Peak Details
Precursor m/z Mass Error (ppm) Retention Time (min) Ion Ratio
205.160 -0.3 12.68 N/A
Formula Finder Results
Name Formula Score m/z (Da) Error (ppm) Error MSMS (ppm) Hit Count
Library Search Results
Name CAS# Formula MM (Da) Fit Rev. Fit Purity CE (eV)

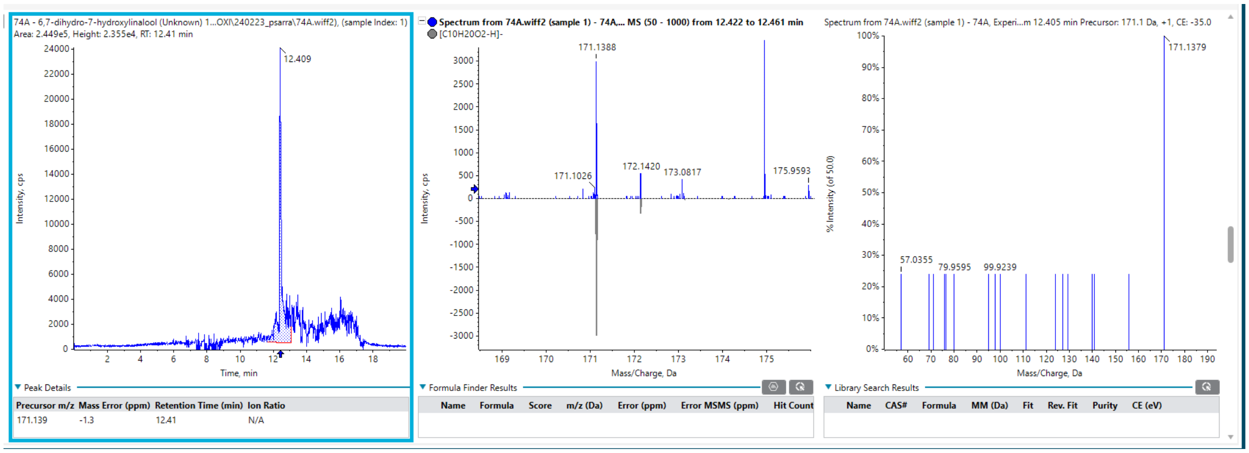
74A - 6,7-dihydro-7-hydroxylinalool (Unknown) 1...OXI\240223_psarra\74A.wiff2), (sample Index: 1)
Area: 2.449e5, Height: 2.355e4, RT: 12.41 min
12.409
Intensity, cps
Time, min
Spectrum from 74A.wiff2 (sample 1) - 74A,... MS (50 - 1000) from 12.422 to 12.461 min
[C10H20O2-H]-
171.1388
171.1026
172.1420
173.0817
175.9593
Mass/Charge, Da
Spectrum from 74A.wiff2 (sample 1) - 74A, Experi...m 12.405 min Precursor: 171.1 Da, +1, CE: -35.0
171.1379
57.0355
79.9595
99.9239
% Intensity (of 50.0)
Peak Details
Precursor m/z Mass Error (ppm) Retention Time (min) Ion Ratio
171.139 -1.3 12.41 N/A
Formula Finder Results
Name Formula Score m/z (Da) Error (ppm) Error MSMS (ppm) Hit Count
Library Search Results
Name CAS# Formula MM (Da) Fit Rev. Fit Purity CE (eV)

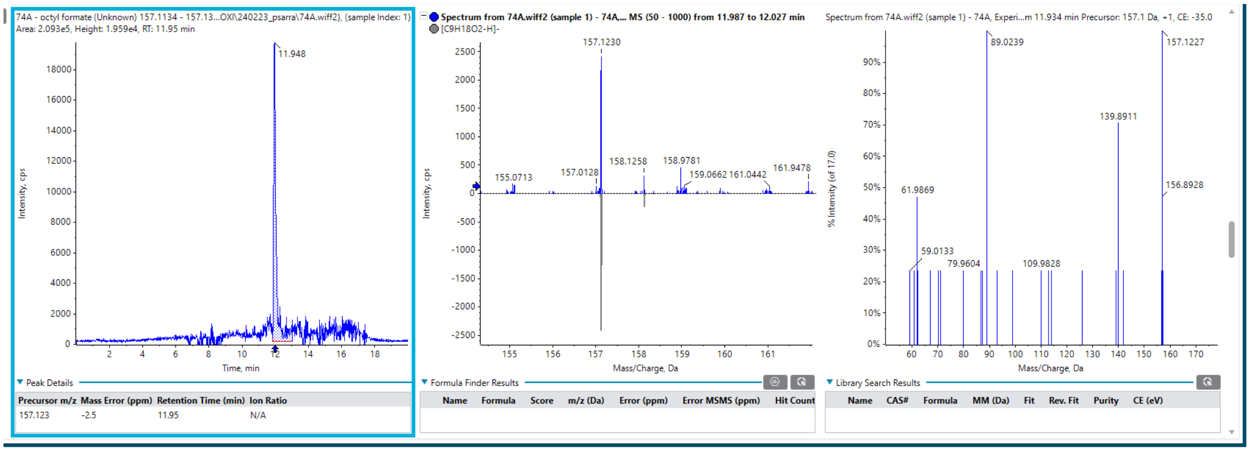
74A - octyl formate (Unknown) 157.1134 - 157.13...OXI\240223_psarra\74A.wiff2), (sample Index: 1)
Area: 2.093e5, Height: 1.959e4, RT: 11.95 min
11.948
Intensity, cps
Time, min
Spectrum from 74A.wiff2 (sample 1) - 74A,... MS (50 - 1000) from 11.987 to 12.027 min
[C9H18O2-H]-
157.1230
155.0713
157.0128
158.1258
158.9781
159.0662
161.0442
161.9478
Mass/Charge, Da
Spectrum from 74A.wiff2 (sample 1) - 74A, Experi...m 11.934 min Precursor: 157.1 Da, +1, CE: -35.0
89.0239
157.1227
139.8911
61.9869
156.8928
59.0133
79.9604
109.9828
% Intensity (of 17.0)
Peak Details
Precursor m/z Mass Error (ppm) Retention Time (min) Ion Ratio
157.123 -2.5 11.95 N/A
Formula Finder Results
Name Formula Score m/z (Da) Error (ppm) Error MSMS (ppm) Hit Count
Library Search Results
Name CAS# Formula MM (Da) Fit Rev. Fit Purity CE (eV)

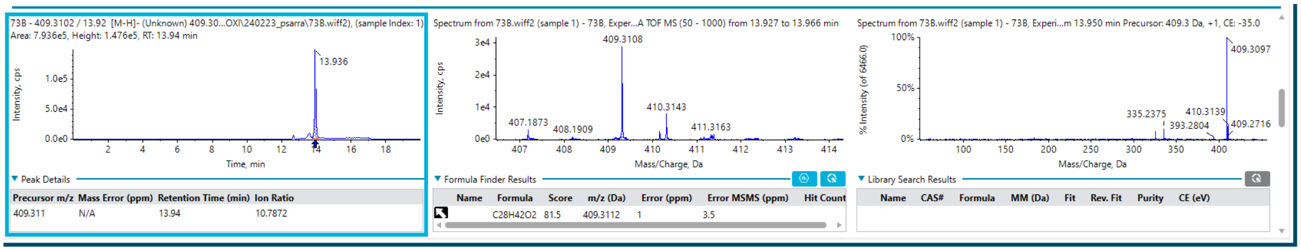
73B - 409.3102 / 13.92 [M-H]- (Unknown) 409.30...OXI\240223_psarra\73B.wiff2), (sample Index: 1)
Area: 7.936e5, Height: 1.476e5, RT: 13.94 min
13.936
Intensity, cps
Time, min
Spectrum from 73B.wiff2 (sample 1) - 73B, Exper...A TOF MS (50 - 1000) from 13.927 to 13.966 min
409.3108
410.3143
407.1873
408.1909
411.3163
Mass/Charge, Da
Spectrum from 73B.wiff2 (sample 1) - 73B, Experi...m 13.950 min Precursor: 409.3 Da, +1, CE: -35.0
409.3097
335.2375
410.3139
393.2804
409.2716
% Intensity (of 6466.0)
Peak Details
Precursor m/z Mass Error (ppm) Retention Time (min) Ion Ratio
409.311 N/A 13.94 10.7872
Formula Finder Results
Name Formula Score m/z (Da) Error (ppm) Error MSMS (ppm) Hit Count
C28H42O2 81.5 409.3112 1 3.5
Library Search Results
Name CAS# Formula MM (Da) Fit Rev. Fit Purity CE (eV)

Figure S1. Extracted Ion Chromatograms (EICs), MS and MS/MS spectra of suspect compounds

Non-target compounds	Chemical classification	Biological activities
**Ethyl 2-acetyl heptanoate**	straight-chain fatty acid	Cardiovascular [126], anti-microbial [127]
**Sesterstatin**	sesterterpenoid	Anti-bacterial [128], anti-cancer [128, 129]
**Cyclohexanecarboxylic acid**	organic compound	Anti-microbial [130], β-oxidation metabolism [131]
**1,2-Hydroxylauric acid**	medium-chain fatty acid	Anti-oxidant, immune activator [132], anti-bacterial [133, 134], anti-cancer [135]
**Dodecanedioic acid**	saturated aliphatic dicarboxylic acid	Anti-cancer, anti-oxidant through mitochondrial pathway [132-135]
**Tisocalcitate**	organic compound (vitamin D derivative)	Cardiovascular, neural development, neuroprotective [136-139]
**Trivalerin**	(ester of valeric acid) straight-chain saturated fatty acid	Anti-inflammatory, neuroprotective [140, 141], anti-oxidant, anti-microbial [142]
**Frangulin B**	anthraquinone	Anti-oxidant, anti-inflammatory, neuroprotective [143], anti-fungal, anti-cancer [144, 145]
**Tetra-tert-butylbiphenol**	aromatic hydrocarbon	Anti-bacterial, anti-microbial [146], anti-inflammatory, anti-hypertensive, anti-cancer [147]

Table S2. Non-target screening results, identified by HPLC-QTOF-MS/MS analysis, in different polarity fractions from *P. lenticonus*/Chios, their chemical classification, and biological activities.

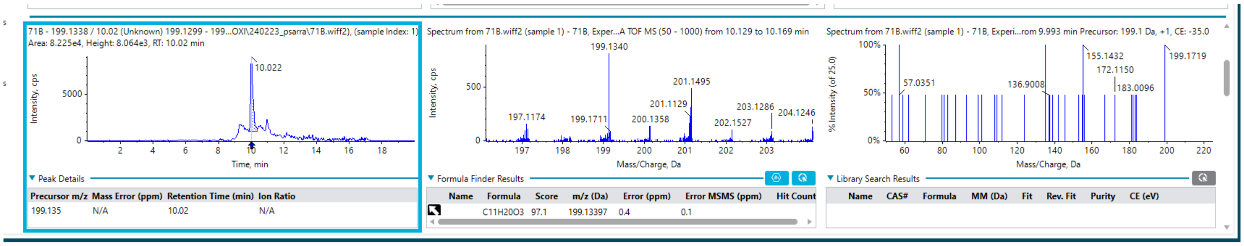
71B - 199.1338 / 10.02 (Unknown) 199.1299 - 199...OXI\240223_psarra\71B.wiff2), (sample Index: 1)
Area: 8.225e4, Height: 8.064e3, RT: 10.02 min
10.022
Intensity, cps
Time, min
Peak Details
Precursor m/z | Mass Error (ppm) | Retention Time (min) | Ion Ratio
199.135 | N/A | 10.02 | N/A
Spectrum from 71B.wiff2 (sample 1) - 71B, Exper...A TOF MS (50 - 1000) from 10.129 to 10.169 min
199.1340
201.1495
201.1129
203.1286
204.1246
197.1174
199.1711
200.1358
202.1527
Mass/Charge, Da
Formula Finder Results
Name | Formula | Score | m/z (Da) | Error (ppm) | Error MSMS (ppm) | Hit Count
C11H20O3 | 97.1 | 199.13397 | 0.4 | 0.1
Spectrum from 71B.wiff2 (sample 1) - 71B, Experi...rom 9.993 min Precursor: 199.1 Da, +1, CE: -35.0
155.1432
199.1719
172.1150
57.0351
136.9008
183.0096
% Intensity (of 25.0)
Mass/Charge, Da
Library Search Results
Name | CAS# | Formula | MM (Da) | Fit | Rev. Fit | Purity | CE (eV)

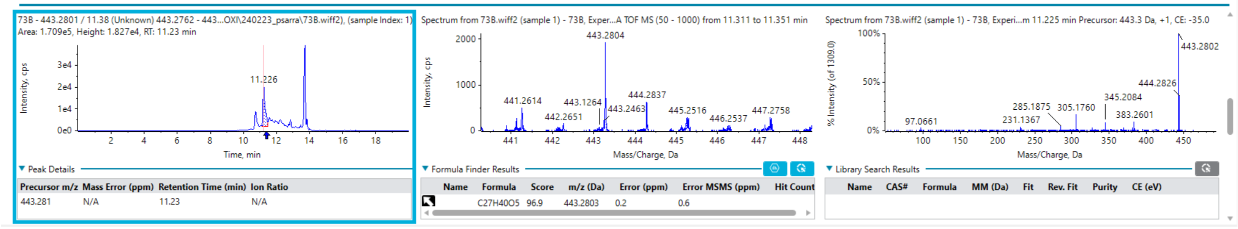
73B - 443.2801 / 11.38 (Unknown) 443.2762 - 443...OXI\240223_psarra\73B.wiff2), (sample Index: 1)
Area: 1.709e5, Height: 1.827e4, RT: 11.23 min
11.226
Intensity, cps
Time, min
Peak Details
Precursor m/z | Mass Error (ppm) | Retention Time (min) | Ion Ratio
443.281 | N/A | 11.23 | N/A
Spectrum from 73B.wiff2 (sample 1) - 73B, Exper...A TOF MS (50 - 1000) from 11.311 to 11.351 min
443.2804
441.2614
443.1264
444.2837
442.2651
443.2463
445.2516
446.2537
447.2758
Mass/Charge, Da
Formula Finder Results
Name | Formula | Score | m/z (Da) | Error (ppm) | Error MSMS (ppm) | Hit Count
C27H40O5 | 96.9 | 443.2803 | 0.2 | 0.6
Spectrum from 73B.wiff2 (sample 1) - 73B, Experi...m 11.225 min Precursor: 443.3 Da, +1, CE: -35.0
443.2802
444.2826
345.2084
285.1875
305.1760
97.0661
231.1367
383.2601
% Intensity (of 1309.0)
Mass/Charge, Da
Library Search Results
Name | CAS# | Formula | MM (Da) | Fit | Rev. Fit | Purity | CE (eV)

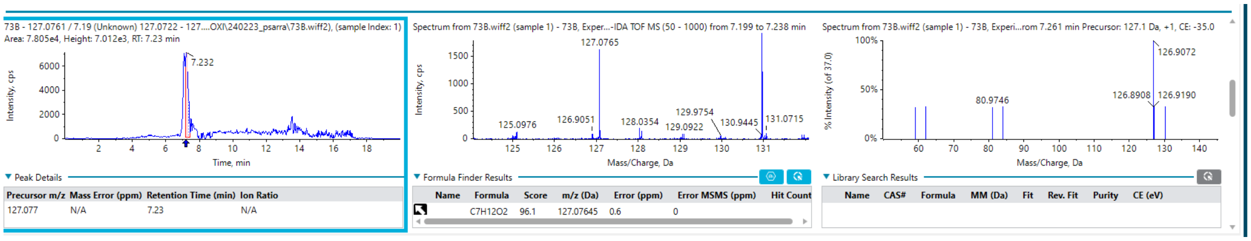
73B - 127.0761 / 7.19 (Unknown) 127.0722 - 127....OXI\240223_psarra\73B.wiff2), (sample Index: 1)
Area: 7.805e4, Height: 7.012e3, RT: 7.23 min
7.232
Intensity, cps
Time, min
Peak Details
Precursor m/z | Mass Error (ppm) | Retention Time (min) | Ion Ratio
127.077 | N/A | 7.23 | N/A
Spectrum from 73B.wiff2 (sample 1) - 73B, Exper...-IDA TOF MS (50 - 1000) from 7.199 to 7.238 min
127.0765
125.0976
126.9051
128.0354
129.9754
129.0922
130.9445
131.0715
Mass/Charge, Da
Formula Finder Results
Name | Formula | Score | m/z (Da) | Error (ppm) | Error MSMS (ppm) | Hit Count
C7H12O2 | 96.1 | 127.07645 | 0.6 | 0
Spectrum from 73B.wiff2 (sample 1) - 73B, Experi...rom 7.261 min Precursor: 127.1 Da, +1, CE: -35.0
126.9072
80.9746
126.8908
126.9190
% Intensity (of 37.0)
Mass/Charge, Da
Library Search Results
Name | CAS# | Formula | MM (Da) | Fit | Rev. Fit | Purity | CE (eV)

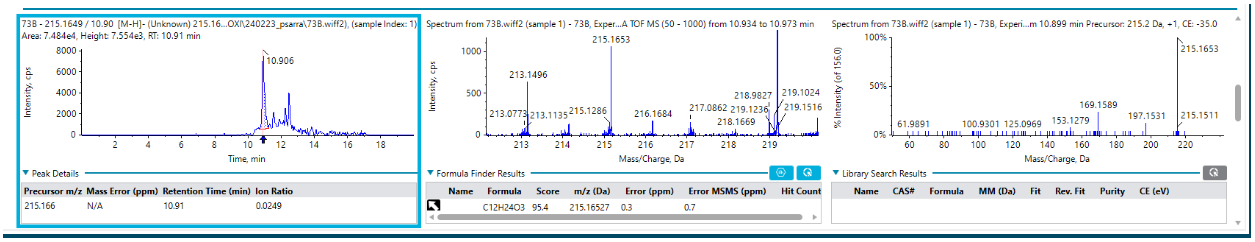
73B - 215.1649 / 10.90 [M-H]- (Unknown) 215.16...OXI\240223_psarra\73B.wiff2), (sample Index: 1)
Area: 7.484e4, Height: 7.554e3, RT: 10.91 min
10.906
Intensity, cps
Time, min
Peak Details
Precursor m/z | Mass Error (ppm) | Retention Time (min) | Ion Ratio
215.166 | N/A | 10.91 | 0.0249
Spectrum from 73B.wiff2 (sample 1) - 73B, Exper...A TOF MS (50 - 1000) from 10.934 to 10.973 min
215.1653
213.1496
218.9827
219.1024
213.0773
213.1135
215.1286
216.1684
217.0862
219.1236
219.1516
218.1669
Mass/Charge, Da
Formula Finder Results
Name | Formula | Score | m/z (Da) | Error (ppm) | Error MSMS (ppm) | Hit Count
C12H24O3 | 95.4 | 215.16527 | 0.3 | 0.7
Spectrum from 73B.wiff2 (sample 1) - 73B, Experi...m 10.899 min Precursor: 215.2 Da, +1, CE: -35.0
215.1653
169.1589
197.1531
215.1511
61.9891
100.9301
125.0969
153.1279
% Intensity (of 156.0)
Mass/Charge, Da
Library Search Results
Name | CAS# | Formula | MM (Da) | Fit | Rev. Fit | Purity | CE (eV)

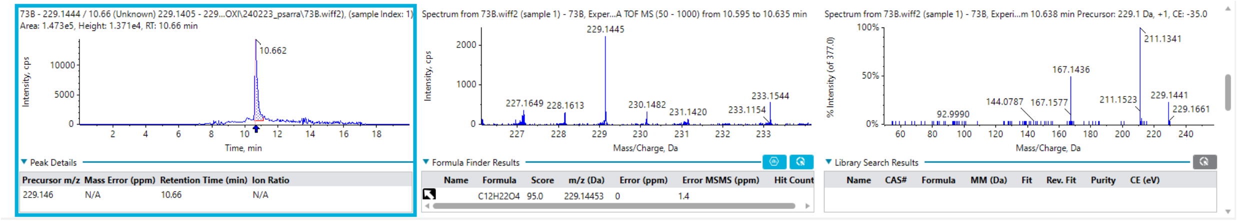
73B - 229.1444 / 10.66 (Unknown) 229.1405 - 229...OXI\240223_psarra\73B.wiff2), (sample Index: 1)
Area: 1.473e5, Height: 1.371e4, RT: 10.66 min
10.662
Intensity, cps
Time, min
Peak Details
Precursor m/z | Mass Error (ppm) | Retention Time (min) | Ion Ratio
229.146 | N/A | 10.66 | N/A
Spectrum from 73B.wiff2 (sample 1) - 73B, Exper...A TOF MS (50 - 1000) from 10.595 to 10.635 min
229.1445
227.1649
228.1613
230.1482
231.1420
233.1544
233.1154
Mass/Charge, Da
Formula Finder Results
Name | Formula | Score | m/z (Da) | Error (ppm) | Error MSMS (ppm) | Hit Count
C12H22O4 | 95.0 | 229.14453 | 0 | 1.4
Spectrum from 73B.wiff2 (sample 1) - 73B, Experi...m 10.638 min Precursor: 229.1 Da, +1, CE: -35.0
211.1341
167.1436
144.0787
167.1577
211.1523
229.1441
229.1661
92.9990
% Intensity (of 377.0)
Mass/Charge, Da
Library Search Results
Name | CAS# | Formula | MM (Da) | Fit | Rev. Fit | Purity | CE (eV)

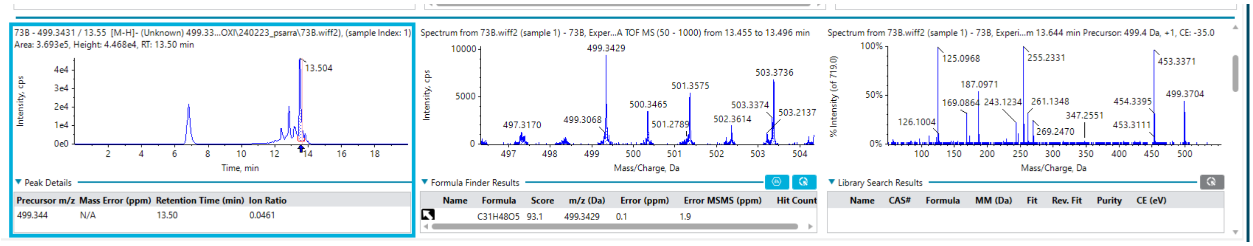
73B - 499.3431 / 13.55 [M-H]- (Unknown) 499.33...OXI\240223_psarra\73B.wiff2), (sample Index: 1)
Area: 3.693e5, Height: 4.468e4, RT: 13.50 min
13.504
Intensity, cps
Time, min
Peak Details
Precursor m/z | Mass Error (ppm) | Retention Time (min) | Ion Ratio
499.344 | N/A | 13.50 | 0.0461
Spectrum from 73B.wiff2 (sample 1) - 73B, Exper...A TOF MS (50 - 1000) from 13.455 to 13.496 min
499.3429
503.3736
501.3575
500.3465
503.3374
502.3614
503.2137
501.2789
497.3170
499.3068
Mass/Charge, Da
Formula Finder Results
Name | Formula | Score | m/z (Da) | Error (ppm) | Error MSMS (ppm) | Hit Count
C31H48O5 | 93.1 | 499.3429 | 0.1 | 1.9
Spectrum from 73B.wiff2 (sample 1) - 73B, Experi...m 13.644 min Precursor: 499.4 Da, +1, CE: -35.0
125.0968
255.2331
453.3371
187.0971
169.0864
243.1234
261.1348
454.3395
499.3704
347.2551
126.1004
453.3111
269.2470
% Intensity (of 719.0)
Mass/Charge, Da
Library Search Results
Name | CAS# | Formula | MM (Da) | Fit | Rev. Fit | Purity | CE (eV)

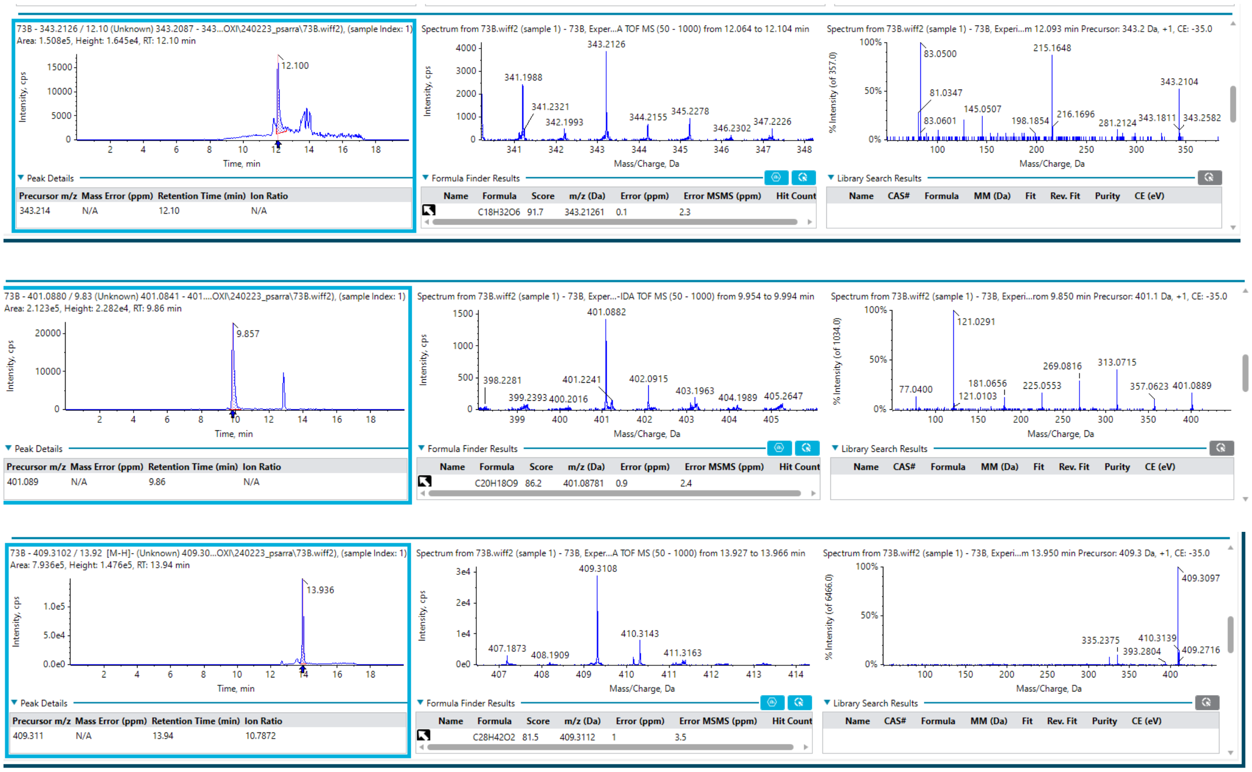

Figure S2. Extracted Ion Chromatograms (EICs), MS and MS/MS spectra of non-target compounds

Tentative Candidate	Hit score	Molecular formula	Hit score
	99.734	$C_{16}H_{24}O_3$	89.955
	98.972	$C_{14}H_{22}O_5$	89.929
Arachidic acid	98.87	$C_{20}H_{40}O_2$	89.862
	98.604	$C_{25}H_{50}N_2O_5$	89.618
	98.543	$C_{16}H_{28}O_4$	89.601
	98.267	$C_{15}H_{22}O_5$	89.549
	97.807	$C_{25}H_{48}N_2O_9S$	89.539
	97.758	$C_{30}H_{55}N_5O_5$	89.517
	97.609	$C_{10}H_{11}NO_6S$	89.475
	97.06	$C_{37}H_{70}N_6O_7S$	89.472
Ethyl 2-acetylheptanoate	97.051	$C_{11}H_{20}O_3$	89.461
	96.945	$C_{16}H_{30}O_2$	89.46
Sesterstatin 7	96.896	$C_{27}H_{40}O_5$,	89.351
	96.536	$C_{18}H_{34}O_3$	89.313
	96.359	$C_{42}H_{77}N_7O_7$	89.311
	96.111	$C_{38}H_{60}O_5S$	89.294
	96.099	$C_{30}H_{48}O_6$	89.28
Cyclohexanecarboxylic acid	96.056	$C_7H_{12}O_2$,	89.268
	96.015	$C_{42}H_{77}N_7O_7$	89.159

	95.972	$C_{22}H_{36}O_4$	89.132
	95.845	$C_{30}H_{50}O_4$	89.126
	95.764	$C_{15}H_{22}O_3$	89.098
	95.733	$C_{31}H_{48}O_5$	89.073
	95.722	$C_{12}H_{22}O_7S$	89.059
	95.438	$C_{46}H_{94}N_{10}O_2S_4$	89.047
	95.316	$C_{18}H_{34}O_5$	88.983
	95.194	$C_8H_{24}N_6O_7S$	88.967
	95.132	$C_{18}H_{32}O_4$	88.872
	95.109	$C_{22}H_{36}O_5$	88.822
Dodecanedioic acid	95.048	$C_{12}H_{22}O_{4,}$	88.75
	94.983	$C_{20}H_{32}N_{10}S$	88.646
1,2-Hydroxylauric acid	94.981	$C_{12}H_{24}O_3$	88.618
	94.964	$C_{11}H_{20}O_4$	88.589
	94.959	$C_{29}H_{46}O_6$	88.514
	94.91	$C_{16}H_{32}O_3$	88.208
	94.833	$C_{14}H_{22}O_2$	88.172
	94.804	$C_{24}H_{46}O_4$	88.085
	94.661	$C_6H_7NO_2S$	88.079
	94.625	$C_{13}H_{22}O_3$	88.05
	94.499	$C_{49}H_{94}N_4O_{10}S$	87.98
	94.485	$C_{15}H_{28}N_6O_2$	87.933
	94.482	$C_{13}H_{20}O_4$	87.888
	94.459	$C_{32}H_{50}O_4$	87.801
	94.389	$C_{16}H_{33}NO_3$	87.761
	94.314	$C_{13}H_{17}NO_5$	87.73
	94.297	$C_{28}H_{40}N_4$	87.706
	93.989	$C_{20}H_{40}N_2O_5$	87.601
	93.825	$C_{15}H_{22}O_2$	87.555
	93.819	$C_{29}H_{46}O_6$	87.526
	93.796	$C_{37}H_{79}N_3O_3S_4$	87.521
	93.602	$C_{20}H_{25}NO_4$	87.518
	93.558	$C_{17}H_{26}N_4O_3$	87.508
	93.548	$C_{18}H_{34}O_3$	87.495
	93.484	$C_{23}H_{48}O_8$	87.469
	93.445	$C_{11}H_{30}N_6O_2S$	87.453
	93.249	$C_{16}H_{30}OS_2$	87.445
	93.208	$C_{36}H_{66}N_6O_6$	87.397
	93.111	$C_{14}H_{22}O_2$	87.389
Tisocalcitate	93.108	$C_{31}H_{48}O_5$	87.284
	93.019	$C_{26}H_{40}O_4$	87.259
	92.99	$C_{29}H_{42}O_5$	86.914
	92.95	$C_{18}H_{34}N_4O_6$	86.81
	92.933	$C_{30}H_{46}O_7$	86.718
	92.926	$C_{36}H_{74}N_2O_5S_2$	86.701
	92.89	$C_{31}H_{48}O_4$	86.668

	92.86	$C_{14}H_{21}NO_3$	86.648
	92.829	$C_{31}H_{52}O_4$	86.558
	92.781	$C_9H_{17}NO_3$	86.555
	92.757	$C_{13}H_{20}O_2$	86.344
	92.663	$C_9H_{10}O_2$	86.332
	92.649	$C_{19}H_{38}N_6O_2S$	86.311
	92.632	$C_{20}H_{34}O_4$	86.186
Frangulin B	92.469	$C_{21}H_{20}O_9$,	86.163
	92.446	$C_{16}H_{26}O_6$	85.957
	92.44	$C_8H_{14}O_4$	85.846
	92.388	$C_{22}H_{38}O_4$	85.776
	92.37	$C_{31}H_{54}O_3$	85.656
	92.322	$C_{17}H_{28}O_5$	85.629
	92.306	$C_{13}H_{24}O_3$	85.616
	92.278	$C_{38}H_{71}N_5O_6S$	85.422
	92.262	$C_{28}H_{46}N_{10}S_2$	85.297
	92.145	$C_{22}H_{40}O_3S_2$	85.292
	92.134	$C_{17}H_{34}O_2$	85.285
	92.098	$C_9H_2N_2O_5S$	85.266
	92.088	$C_{14}H_{28}O_2$	85.204
	92.05	$C_{10}H_{13}NO_3$	85.187
	91.984	$C_{24}H_{36}N_4O_3$	85.069
	91.957	$C_{28}H_{41}N_7O_4$	84.998
	91.938	$C_{10}H_{18}O_4$	84.822
	91.935	$C_{17}H_{14}N_6$	84.743
	91.761	$C_{15}H_{28}O_4$	84.691
	91.754	$C_{21}H_{34}O_4$	84.638
	91.74	$C_{10}H_{20}O_3$	84.617
(E)-4-Methoxycinnamic acid	91.727	$C_{10}H_{10}O_3$,	84.307
	91.724	$C_{12}H_{20}O_2$	84.088
Trivalerin	91.708	$C_{18}H_{32}O_6$,	83.958
	91.681	$C_9H_{18}O_3$	83.809
	91.643	$C_{18}H_{32}O_6S$	83.602
	91.583	$C_{18}H_{36}O_4$	83.471
	91.467	$C_{29}H_{47}NO_3$	83.465
	91.417	$C_{10}H_{20}O_2$	83.198
	91.283	$C_{12}H_{24}O_2$	83.089
	91.214	$C_{14}H_{28}O_3$	83.05
	91.183	$C_{13}H_{25}NO_3$	83.024
	91.179	$C_{11}H_{14}O_3$	82.927
	91.177	$C_{25}H_{59}N_9O_6S$	82.724
	91.091	$C_{30}H_{50}O_5$	82.716
	90.985	$C_{30}H_{46}O_4$	82.406
	90.984	$C_{22}H_{42}O_4$	82.331
	90.763	$C_{35}H_{60}O_3$	82.331
	90.723	$C_9H_{26}N_6O_2S$	82.214

	90.704	$C_{30}H_{38}N_2O_3$	81.911
4,4',6,6'-Tetra-tert-butyl-2,2'-biphenol	90.682	$C_{28}H_{42}O_2$	81.535
	90.674	$C_{29}H_{46}O_4$	81.506
	90.612	$C_{18}H_{36}O_3$	81.445
	90.589	$C_{24}H_{45}N_5O_7$	81.376
	90.512	$C_{12}H_{26}O_4S$	81.038
	90.464	$C_{24}H_{33}NO_4$	80.78
	90.377	$C_{30}H_{48}O_3$	80.777
	90.376	$C_{18}H_{34}O_2$	80.428
	90.355	$C_{18}H_{36}O_2$	80.333
	90.235	$C_3H_6N_{10}O_7$	80.312
	90.083	$C_{19}H_{42}N_{10}OS$	80.034
	90.028	$C_{32}H_{44}O_2$	80.028

Table S3. Unequivocal molecular formulas and hit scores of non-target compounds identified in apolar, medium polar and polar fractions from *P. lenticonus*/Chios assigned by SCIEX OS software and the SCIEX Natural Products Library

References

1. Kalousi, F.D., et al., Regulation of Energy Metabolism and Anti-Inflammatory Activities of Mastiha Fractions from Pistacia lentiscus L. var. chia. Foods, 2023. 12(7).
2. Pisha, E., et al., Discovery of betulinic acid as a selective inhibitor of human melanoma that functions by induction of apoptosis. Nat Med, 1995. 1(10): p. 1046-51.
3. Fulda, S., Betulinic acid: a natural product with anticancer activity. Mol Nutr Food Res, 2009. 53(1): p. 140-6.
4. Lou, H., et al., A Review on Preparation of Betulinic Acid and Its Biological Activities. Molecules, 2021. 26(18).
5. Costa, J.F., et al., Potent anti-inflammatory activity of betulinic acid treatment in a model of lethal endotoxemia. Int Immunopharmacol, 2014. 23(2): p. 469-74.
6. Armah, F.A., et al., Erythroivorensin: A novel anti-inflammatory diterpene from the root-bark of Erythrophleum ivorense (A Chev.). Fitoterapia, 2015. 105: p. 37-42.
7. Kim, S.J., et al., Beneficial effect of betulinic acid on hyperglycemia via suppression of hepatic glucose production. J Agric Food Chem, 2014. 62(2): p. 434-42.
8. Yoon, J.J., et al., Protective effect of betulinic acid on early atherosclerosis in diabetic apolipoprotein-E gene knockout mice. Eur J Pharmacol, 2017. 796: p. 224-232.
9. Dong, Y., et al., Coniferaldehyde attenuates Alzheimer's pathology via activation of Nrf2 and its targets. Theranostics, 2020. 10(1): p. 179-200.
10. Kim, K.M., et al., Coniferaldehyde inhibits LPS-induced apoptosis through the PKC alpha/beta II/Nrf-2/HO-1 dependent pathway in RAW264.7 macrophage cells. Environ Toxicol Pharmacol, 2016. 48: p. 85-93.
11. Gai, H., et al., Coniferaldehyde ameliorates the lipid and glucose metabolism in palmitic acid-induced HepG2 cells via the LKB1/AMPK signaling pathway. J Food Sci, 2020. 85(11): p. 4050-4060.

12. Dai, W., et al., Euscaphic acid inhibits proliferation and promotes apoptosis of nasopharyngeal carcinoma cells by silencing the PI3K/AKT/mTOR signaling pathway. Am J Transl Res, 2019. 11(4): p. 2090-2098.
13. Jeong, N.H., et al., Inhibitory Effects of Euscaphic Acid in the Atopic Dermatitis Model by Reducing Skin Inflammation and Intense Pruritus. Inflammation, 2022. 45(4): p. 1680-1691.
14. Shi, C., et al., Euscaphic acid and Tormentic acid protect vascular endothelial cells against hypoxia-induced apoptosis via PI3K/AKT or ERK 1/2 signaling pathway. Life Sci, 2020. 252: p. 117666.
15. Jayaprakasha, G.K., L. Jaganmohan Rao, and K.K. Sakariah, Antioxidant activities of flavidin in different in vitro model systems. Bioorg Med Chem, 2004. 12(19): p. 5141-6.
16. Ysrafil, Y., et al., Anti-inflammatory activities of flavonoid derivates. ADMET DMPK, 2023. 11(3): p. 331-359.
17. Al-Khayri, J.M., et al., Flavonoids as Potential Anti-Inflammatory Molecules: A Review. Molecules, 2022. 27(9).
18. Wang, K.L., Y.C. Yu, and S.M. Hsia, Perspectives on the Role of Isoliquiritigenin in Cancer. Cancers (Basel), 2021. 13(1).
19. Hirchaud, F., et al., Isoliquiritigenin induces caspase-dependent apoptosis via downregulation of HPV16 E6 expression in cervical cancer Ca Ski cells. Planta Med, 2013. 79(17): p. 1628-35.
20. Caporali, S., et al., Anti-Inflammatory and Active Biological Properties of the Plant-Derived Bioactive Compounds Luteolin and Luteolin 7-Glucoside. Nutrients, 2022. 14(6).
21. Samec, M., et al., Flavonoids against the Warburg phenotype-concepts of predictive, preventive and personalised medicine to cut the Gordian knot of cancer cell metabolism. EPMA J, 2020. 11(3): p. 377-398.
22. Kang, K.A., et al., Luteolin induces apoptotic cell death via antioxidant activity in human colon cancer cells. Int J Oncol, 2017. 51(4): p. 1169-1178.
23. Kou, J.J., et al., Luteolin alleviates cognitive impairment in Alzheimer's disease mouse model via inhibiting endoplasmic reticulum stress-dependent neuroinflammation. Acta Pharmacol Sin, 2022. 43(4): p. 840-849.
24. de Araujo Junior, R.F., et al., Ceramide and palmitic acid inhibit macrophage-mediated epithelial-mesenchymal transition in colorectal cancer. Mol Cell Biochem, 2020. 468(1-2): p. 153-168.
25. Yu, G., et al., Loss of p53 Sensitizes Cells to Palmitic Acid-Induced Apoptosis by Reactive Oxygen Species Accumulation. Int J Mol Sci, 2019. 20(24).
26. Mancini, A., et al., Biological and Nutritional Properties of Palm Oil and Palmitic Acid: Effects on Health. Molecules, 2015. 20(9): p. 17339-61.
27. Innis, S.M., Palmitic Acid in Early Human Development. Crit Rev Food Sci Nutr, 2016. 56(12): p. 1952-9.
28. Acosta-Montano, P., et al., Fatty Acid and Lipopolysaccharide Effect on Beta Cells Proteostasis and its Impact on Insulin Secretion. Cells, 2019. 8(8).
29. Liu, Z., et al., Emerging roles of protein palmitoylation and its modifying enzymes in cancer cell signal transduction and cancer therapy. Int J Biol Sci, 2022. 18(8): p. 3447-3457.
30. Libran-Perez, M., et al., Antiviral activity of palmitic acid via autophagic flux inhibition in zebrafish (Danio rerio). Fish Shellfish Immunol, 2019. 95: p. 595-605.
31. Syed, I., et al., Palmitic Acid Hydroxystearic Acids Activate GPR40, Which Is Involved in Their Beneficial Effects on Glucose Homeostasis. Cell Metab, 2018. 27(2): p. 419-427 e4.
32. Hammarstedt, A., et al., Adipose tissue dysfunction is associated with low levels of the novel Palmitic Acid Hydroxystearic Acids. Sci Rep, 2018. 8(1): p. 15757.
33. Kamata, S., et al., PPARalpha Ligand-Binding Domain Structures with Endogenous Fatty Acids and Fibrates. iScience, 2020. 23(11): p. 101727.

34. Yore, M.M., et al., Discovery of a class of endogenous mammalian lipids with anti-diabetic and anti-inflammatory effects. Cell, 2014. 159(2): p. 318-32.
35. Wang, Z.J., et al., Neuroprotective effects of stearic acid against toxicity of oxygen/glucose deprivation or glutamate on rat cortical or hippocampal slices. Acta Pharmacol Sin, 2006. 27(2): p. 145-50.
36. Khan, A.A., et al., Design, synthesis and in vitro anticancer evaluation of a stearic acid-based ester conjugate. Anticancer Res, 2013. 33(6): p. 2517-24.
37. Fekete, K., et al., Long-chain polyunsaturated fatty acid status in obesity: a systematic review and meta-analysis. Obes Rev, 2015. 16(6): p. 488-97.
38. Hodson, L., C.M. Skeaff, and B.A. Fielding, Fatty acid composition of adipose tissue and blood in humans and its use as a biomarker of dietary intake. Prog Lipid Res, 2008. 47(5): p. 348-80.
39. Ren, J. and S.H. Chung, Anti-inflammatory effect of alpha-linolenic acid and its mode of action through the inhibition of nitric oxide production and inducible nitric oxide synthase gene expression via NF-kappaB and mitogen-activated protein kinase pathways. J Agric Food Chem, 2007. 55(13): p. 5073-80.
40. Fan, N., J.L. Fusco, and D.W. Rosenberg, Antioxidant and Anti-Inflammatory Properties of Walnut Constituents: Focus on Personalized Cancer Prevention and the Microbiome. Antioxidants (Basel), 2023. 12(5).
41. Yan, H., et al., The Antitumor Effects of alpha-Linolenic Acid. J Pers Med, 2024. 14(3).
42. Azrad, M., C. Turgeon, and W. Demark-Wahnefried, Current evidence linking polyunsaturated Fatty acids with cancer risk and progression. Front Oncol, 2013. 3: p. 224.
43. Vieira, C., et al., Effect of ricinoleic acid in acute and subchronic experimental models of inflammation. Mediators Inflamm, 2000. 9(5): p. 223-8.
44. Pabis, S. and J. Kula, Synthesis and Bioactivity of (R)-Ricinoleic Acid Derivatives: A Review. Curr Med Chem, 2016. 23(35): p. 4037-4056.
45. Minto, R.E. and B.J. Blacklock, Biosynthesis and function of polyacetylenes and allied natural products. Prog Lipid Res, 2008. 47(4): p. 233-306.
46. Xu, T., et al., A potent plant-derived antifungal acetylenic acid mediates its activity by interfering with fatty acid homeostasis. Antimicrob Agents Chemother, 2012. 56(6): p. 2894-907.
47. Rengachar, P., et al., Gamma-Linolenic Acid (GLA) Protects against Ionizing Radiation-Induced Damage: An In Vitro and In Vivo Study. Biomolecules, 2022. 12(6).
48. Das, U.N. and K.P. Rao, Effect of gamma-linolenic acid and prostaglandins E1 on gamma-radiation and chemical-induced genetic damage to the bone marrow cells of mice. Prostaglandins Leukot Essent Fatty Acids, 2006. 74(3): p. 165-73.
49. Leaver, H.A., et al., Highly unsaturated fatty acid induced tumour regression in glioma pharmacodynamics and bioavailability of gamma linolenic acid in an implantation glioma model: effects on tumour biomass, apoptosis and neuronal tissue histology. Prostaglandins Leukot Essent Fatty Acids, 2002. 67(5): p. 283-92.
50. Das, U.N., Gamma-linolenic acid therapy of human glioma-a review of in vitro, in vivo, and clinical studies. Med Sci Monit, 2007. 13(7): p. RA119-31.
51. Andreoli Miyake, J., R. Nascimento Gomes, and A. Colquhoun, Gamma-Linolenic acid alters migration, proliferation and apoptosis in human and rat glioblastoma cells. Prostaglandins Other Lipid Mediat, 2020. 150: p. 106452.
52. Benadiba, M., J.A. Miyake, and A. Colquhoun, Gamma-linolenic acid alters Ku80, E2F1, and bax expression and induces micronucleus formation in C6 glioma cells in vitro. IUBMB Life, 2009. 61(3): p. 244-51.
53. Miyake, J.A., M. Benadiba, and A. Colquhoun, Gamma-linolenic acid inhibits both tumour cell cycle progression and angiogenesis in the orthotopic C6 glioma model through changes in VEGF,

Flt1, ERK1/2, MMP2, cyclin D1, pRb, p53 and p27 protein expression. Lipids Health Dis, 2009. 8: p. 8.
54. Oepen, K., et al., Myristic Acid Inhibits the Activity of the Bacterial ABC Transporter BmrA. Int J Mol Sci, 2021. 22(24).
55. Contreras, C.M., et al., Myristic acid produces anxiolytic-like effects in Wistar rats in the elevated plus maze. Biomed Res Int, 2014. 2014: p. 492141.
56. Javid, S., et al., Discovery of Novel Myristic Acid Derivatives as N-Myristoyltransferase Inhibitors: Design, Synthesis, Analysis, Computational Studies and Antifungal Activity. Antibiotics (Basel), 2023. 12(7).
57. Prasath, K.G., et al., Anti-inflammatory potential of myristic acid and palmitic acid synergism against systemic candidiasis in Danio rerio (Zebrafish). Biomed Pharmacother, 2021. 133: p. 111043.
58. Alonso-Castro, A.J., et al., Myristic acid reduces skin inflammation and nociception. J Food Biochem, 2022. 46(1): p. e14013.
59. Popeijus, H.E., et al., Fatty acid chain length and saturation influences PPARalpha transcriptional activation and repression in HepG2 cells. Mol Nutr Food Res, 2014. 58(12): p. 2342-9.
60. Pompeia, C., et al., Effect of fatty acids on leukocyte function. Braz J Med Biol Res, 2000. 33(11): p. 1255-68.
61. Brash, A.R., Arachidonic acid as a bioactive molecule. J Clin Invest, 2001. 107(11): p. 1339-45.
62. Tallima, H. and R. El Ridi, Arachidonic acid: Physiological roles and potential health benefits - A review. J Adv Res, 2018. 11: p. 33-41.
63. Tokuda, H., et al., Differential effect of arachidonic acid and docosahexaenoic acid on age-related decreases in hippocampal neurogenesis. Neurosci Res, 2014. 88: p. 58-66.
64. Fukaya, T., et al., Arachidonic acid preserves hippocampal neuron membrane fluidity in senescent rats. Neurobiol Aging, 2007. 28(8): p. 1179-86.
65. Antollini, S.S. and F.J. Barrantes, Fatty Acid Regulation of Voltage- and Ligand-Gated Ion Channel Function. Front Physiol, 2016. 7: p. 573.
66. Wang, Z.J., et al., Neuroprotective effects of arachidonic acid against oxidative stress on rat hippocampal slices. Chem Biol Interact, 2006. 163(3): p. 207-17.
67. Perez, R., et al., Blockade of arachidonic acid incorporation into phospholipids induces apoptosis in U937 promonocytic cells. J Lipid Res, 2006. 47(3): p. 484-91.
68. Trostchansky, A., I. Wood, and H. Rubbo, Regulation of arachidonic acid oxidation and metabolism by lipid electrophiles. Prostaglandins Other Lipid Mediat, 2021. 152: p. 106482.
69. Trostchansky, A. and H. Rubbo, Anti-inflammatory signaling actions of electrophilic nitro-arachidonic acid in vascular cells and astrocytes. Arch Biochem Biophys, 2017. 617: p. 155-161.
70. Sala, A., et al., Two-pronged approach to anti-inflammatory therapy through the modulation of the arachidonic acid cascade. Biochem Pharmacol, 2018. 158: p. 161-173.
71. Wang, T., et al., Arachidonic Acid Metabolism and Kidney Inflammation. Int J Mol Sci, 2019. 20(15).
72. Wang, Y., et al., Arachidonic acid epoxygenase metabolites stimulate endothelial cell growth and angiogenesis via mitogen-activated protein kinase and phosphatidylinositol 3-kinase/Akt signaling pathways. J Pharmacol Exp Ther, 2005. 314(2): p. 522-32.
73. Li, N., et al., Use of metabolomic profiling in the study of arachidonic acid metabolism in cardiovascular disease. Congest Heart Fail, 2011. 17(1): p. 42-6.
74. Sonnweber, T., et al., Arachidonic Acid Metabolites in Cardiovascular and Metabolic Diseases. Int J Mol Sci, 2018. 19(11).
75. Tavolari, S., et al., Licofelone, a dual COX/5-LOX inhibitor, induces apoptosis in HCA-7 colon cancer cells through the mitochondrial pathway independently from its ability to affect the arachidonic acid cascade. Carcinogenesis, 2008. 29(2): p. 371-80.

76. Dai, D., et al., Polymorphisms in human CYP2C8 decrease metabolism of the anticancer drug paclitaxel and arachidonic acid. Pharmacogenetics, 2001. 11(7): p. 597-607.
77. Colombero, C., et al., Cytochrome 450 metabolites of arachidonic acid (20-HETE, 11,12-EET and 14,15-EET) promote pheochromocytoma cell growth and tumor associated angiogenesis. Biochimie, 2020. 171-172: p. 147-157.
78. Shao, J., et al., Involvement of the arachidonic acid cytochrome P450 epoxygenase pathway in the proliferation and invasion of human multiple myeloma cells. PeerJ, 2016. 4: p. e1925.
79. Pozzi, A., et al., The anti-tumorigenic properties of peroxisomal proliferator-activated receptor alpha are arachidonic acid epoxygenase-mediated. J Biol Chem, 2010. 285(17): p. 12840-50.
80. Venn-Watson, S., R. Lumpkin, and E.A. Dennis, Efficacy of dietary odd-chain saturated fatty acid pentadecanoic acid parallels broad associated health benefits in humans: could it be essential? Sci Rep, 2020. 10(1): p. 8161.
81. Shafaghat, A., Antioxidant, antimicrobial activities and fatty acid components of flower, leaf, stem and seed of Hypericum scabrum. Nat Prod Commun, 2011. 6(11): p. 1739-42.
82. Singh, D., et al., Anti-Inflammatory Effect of Dietary Pentadecanoic Fatty Acid Supplementation on Inflammatory Bowel Disease in SAMP1/YitFc Mice. Nutrients, 2024. 16(17).
83. To, N.B., et al., Pentadecanoic Acid, an Odd-Chain Fatty Acid, Suppresses the Stemness of MCF-7/SC Human Breast Cancer Stem-Like Cells through JAK2/STAT3 Signaling. Nutrients, 2020. 12(6).
84. To, N.B., et al., Effects of Combined Pentadecanoic Acid and Tamoxifen Treatment on Tamoxifen Resistance in MCF-7/SC Breast Cancer Cells. Int J Mol Sci, 2022. 23(19).
85. Wang, X., et al., Antioxidant activities of oleanolic acid in vitro: possible role of Nrf2 and MAP kinases. Chem Biol Interact, 2010. 184(3): p. 328-37.
86. Wang, X., et al., Oleanolic acid improves hepatic insulin resistance via antioxidant, hypolipidemic and anti-inflammatory effects. Mol Cell Endocrinol, 2013. 376(1-2): p. 70-80.
87. Dzubak, P., et al., Pharmacological activities of natural triterpenoids and their therapeutic implications. Nat Prod Rep, 2006. 23(3): p. 394-411.
88. Allouche, Y., et al., Antioxidant, antiproliferative, and pro-apoptotic capacities of pentacyclic triterpenes found in the skin of olives on MCF-7 human breast cancer cells and their effects on DNA damage. J Agric Food Chem, 2011. 59(1): p. 121-30.
89. Du, Y. and K.M. Ko, Oleanolic acid protects against myocardial ischemia-reperfusion injury by enhancing mitochondrial antioxidant mechanism mediated by glutathione and alpha-tocopherol in rats. Planta Med, 2006. 72(3): p. 222-7.
90. Tang, Z.Y., et al., Anticancer activity of oleanolic acid and its derivatives: Recent advances in evidence, target profiling and mechanisms of action. Biomed Pharmacother, 2022. 145: p. 112397.
91. Gupta, S., et al., Cytotoxic evaluation of semisynthetic ester and amide derivatives of oleanolic acid. Nat Prod Commun, 2010. 5(10): p. 1567-70.
92. Roohbakhsh, A., et al., Molecular mechanisms behind the biological effects of hesperidin and hesperetin for the prevention of cancer and cardiovascular diseases. Life Sci, 2015. 124: p. 64-74.
93. Parhiz, H., et al., Antioxidant and anti-inflammatory properties of the citrus flavonoids hesperidin and hesperetin: an updated review of their molecular mechanisms and experimental models. Phytother Res, 2015. 29(3): p. 323-31.
94. Ferreira de Oliveira, J.M.P., C. Santos, and E. Fernandes, Therapeutic potential of hesperidin and its aglycone hesperetin: Cell cycle regulation and apoptosis induction in cancer models. Phytomedicine, 2020. 73: p. 152887.
95. Fontana, A., B. Spolaore, and P. Polverino de Laureto, The biological activities of protein/oleic acid complexes reside in the fatty acid. Biochim Biophys Acta, 2013. 1834(6): p. 1125-43.
96. Carrillo, C., M. Cavia Mdel, and S.R. Alonso-Torre, Antitumor effect of oleic acid; mechanisms of action: a review. Nutr Hosp, 2012. 27(6): p. 1860-5.

97. Carrillo, C., M. Cavia Mdel, and S. Alonso-Torre, Role of oleic acid in immune system; mechanism of action; a review. Nutr Hosp, 2012. 27(4): p. 978-90.
98. Masner, M., et al., Linoleic and oleic acids enhance cell migration by altering the dynamics of microtubules and the remodeling of the actin cytoskeleton at the leading edge. Sci Rep, 2021. 11(1): p. 14984.
99. Benjamin, S. and F. Spener, Conjugated linoleic acids as functional food: an insight into their health benefits. Nutr Metab (Lond), 2009. 6: p. 36.
100. Kolar, M.J., et al., Linoleic acid esters of hydroxy linoleic acids are anti-inflammatory lipids found in plants and mammals. J Biol Chem, 2019. 294(27): p. 10698-10707.
101. Cahoon, E.B. and Y. Li-Beisson, Plant unusual fatty acids: learning from the less common. Curr Opin Plant Biol, 2020. 55: p. 66-73.
102. Scott, S., E.B. Cahoon, and L. Busta, Variation on a theme: the structures and biosynthesis of specialized fatty acid natural products in plants. Plant J, 2022. 111(4): p. 954-965.
103. Lin, W.C., et al., Octanoic acid promotes clearance of antibiotic-tolerant cells and eradicates biofilms of Staphylococcus aureus isolated from recurrent bovine mastitis. Biofilm, 2023. 6: p. 100149.
104. Zhang, H., et al., Antimicrobial action of octanoic acid against Escherichia coli O157:H7 during washing of baby spinach and grape tomatoes. Food Res Int, 2019. 125: p. 108523.
105. Charlot, A., et al., Octanoic Acid-Enrichment Diet Improves Endurance Capacity and Reprograms Mitochondrial Biogenesis in Skeletal Muscle of Mice. Nutrients, 2022. 14(13).
106. Maczka, W., et al., Natural Compounds in the Battle against Microorganisms-Linalool. Molecules, 2022. 27(20).
107. Pereira, I., et al., Linalool bioactive properties and potential applicability in drug delivery systems. Colloids Surf B Biointerfaces, 2018. 171: p. 566-578.
108. Liu, X., et al., Antibacterial activity and mechanism of linalool against Pseudomonas aeruginosa. Microb Pathog, 2020. 141: p. 103980.
109. Elbe, H., et al., Anticancer activity of linalool: comparative investigation of ultrastructural changes and apoptosis in breast cancer cells. Ultrastruct Pathol, 2022. 46(4): p. 348-358.
110. Zielinska-Blajet, M. and J. Feder-Kubis, Monoterpenes and Their Derivatives-Recent Development in Biological and Medical Applications. Int J Mol Sci, 2020. 21(19).
111. Koziol, A., et al., An overview of the pharmacological properties and potential applications of natural monoterpenes. Mini Rev Med Chem, 2014. 14(14): p. 1156-68.
112. Khusnutdinova, E.F., et al., Development of New Antimicrobial Oleanonic Acid Polyamine Conjugates. Antibiotics (Basel), 2022. 11(1).
113. Castellano, J.M., S. Ramos-Romero, and J.S. Perona, Oleanolic Acid: Extraction, Characterization and Biological Activity. Nutrients, 2022. 14(3).
114. Vuorinen, A., et al., Pistacia lentiscus Oleoresin: Virtual Screening and Identification of Masticadienonic and Isomasticadienonic Acids as Inhibitors of 11beta-Hydroxysteroid Dehydrogenase 1. Planta Med, 2015. 81(6): p. 525-32.
115. Assimopoulou, A.N. and V.P. Papageorgiou, GC-MS analysis of penta- and tetra-cyclic triterpenes from resins of Pistacia species. Part I. Pistacia lentiscus var. Chia. Biomed Chromatogr, 2005. 19(4): p. 285-311.
116. Meng, Q., et al., Biological function of resveratrol and its application in animal production: a review. J Anim Sci Biotechnol, 2023. 14(1): p. 25.
117. Bhat, K.P.L., J.W. Kosmeder, 2nd, and J.M. Pezzuto, Biological effects of resveratrol. Antioxid Redox Signal, 2001. 3(6): p. 1041-64.
118. Yu, X., Y. Jia, and F. Ren, Multidimensional biological activities of resveratrol and its prospects and challenges in the health field. Front Nutr, 2024. 11: p. 1408651.

119. Santos, J.A., et al., Resveratrol and analogues: a review of antioxidant activity and applications to human health. Recent Pat Food Nutr Agric, 2013. 5(2): p. 144-53.
120. Plowuszynska, A. and A. Gliszczynska, Recent Developments in Therapeutic and Nutraceutical Applications of p-Methoxycinnamic Acid from Plant Origin. Molecules, 2021. 26(13).
121. Rychlicka, M., A. Rot, and A. Gliszczynska, Biological Properties, Health Benefits and Enzymatic Modifications of Dietary Methoxylated Derivatives of Cinnamic Acid. Foods, 2021. 10(6).
122. Adisakwattana, S., Cinnamic Acid and Its Derivatives: Mechanisms for Prevention and Management of Diabetes and Its Complications. Nutrients, 2017. 9(2).
123. Yoon, B.K., et al., Antibacterial Free Fatty Acids and Monoglycerides: Biological Activities, Experimental Testing, and Therapeutic Applications. Int J Mol Sci, 2018. 19(4).
124. Kim, J.J. and H.K. Kim, Antioxidant and Antibacterial Activity of Caprylic Acid Vanillyl Ester Produced by Lipase-Mediated Transesterification. J Microbiol Biotechnol, 2021. 31(2): p. 317-326.
125. Altinoz, M.A., A. Ozpinar, and T.N. Seyfried, Caprylic (Octanoic) Acid as a Potential Fatty Acid Chemotherapeutic for Glioblastoma. Prostaglandins Leukot Essent Fatty Acids, 2020. 159: p. 102142.
126. Okere, I.C., et al., Differential effects of heptanoate and hexanoate on myocardial citric acid cycle intermediates following ischemia-reperfusion. J Appl Physiol (1985), 2006. 100(1): p. 76-82.
127. Huang, C.B., et al., Short- and medium-chain fatty acids exhibit antimicrobial activity for oral microorganisms. Arch Oral Biol, 2011. 56(7): p. 650-4.
128. Wisetsai, A., et al., Scalarane Sesterterpenoids with Antibacterial and Anti-Proliferative Activities from the Mushroom Neonothopanus nambi. Molecules, 2021. 26(24).
129. Zhang, C. and Y. Liu, Targeting cancer with sesterterpenoids: the new potential antitumor drugs. J Nat Med, 2015. 69(3): p. 255-66.
130. Jarboe, L.R., L.A. Royce, and P. Liu, Understanding biocatalyst inhibition by carboxylic acids. Front Microbiol, 2013. 4: p. 272.
131. Kallscheuer, N., et al., Reversal of beta-oxidative pathways for the microbial production of chemicals and polymer building blocks. Metab Eng, 2017. 42: p. 33-42.
132. Zhang, B., et al., Comparison of lauric acid and 12-hydroxylauric acid in the alleviation of drought stress in peach (Prunus persica (L.) Batsch). Front Plant Sci, 2022. 13: p. 1025569.
133. Deen, A., et al., Chemical composition and health benefits of coconut oil: an overview. J Sci Food Agric, 2021. 101(6): p. 2182-2193.
134. Liang, C., et al., Lauric Acid Is a Potent Biological Control Agent That Damages the Cell Membrane of Phytophthora sojae. Front Microbiol, 2021. 12: p. 666761.
135. Takagi, T., et al., Lauric Acid Overcomes Hypoxia-Induced Gemcitabine Chemoresistance in Pancreatic Ductal Adenocarcinoma. Int J Mol Sci, 2023. 24(8).
136. Aihara, K., et al., Disruption of nuclear vitamin D receptor gene causes enhanced thrombogenicity in mice. J Biol Chem, 2004. 279(34): p. 35798-802.
137. Pilz, S., et al., Vitamin D supplementation: a promising approach for the prevention and treatment of strokes. Curr Drug Targets, 2011. 12(1): p. 88-96.
138. Wang, T.J., et al., Vitamin D deficiency and risk of cardiovascular disease. Circulation, 2008. 117(4): p. 503-11.
139. Pilkey, N.G., et al., Does Native Vitamin D Supplementation Have Pleiotropic Effects in Patients with End-Stage Kidney Disease? A Systematic Review of Randomized Trials. Nutrients, 2023. 15(13).
140. Nguyen, T.D., et al., Monovalerin and trivalerin increase brain acetic acid, decrease liver succinic acid, and alter gut microbiota in rats fed high-fat diets. Eur J Nutr, 2019. 58(4): p. 1545-1560.

141. Nguyen, T.D., et al., Monobutyrin and Monovalerin Affect Brain Short-Chain Fatty Acid Profiles and Tight-Junction Protein Expression in ApoE-Knockout Rats Fed High-Fat Diets. Nutrients, 2020. 12(4).
142. Onrust, L., et al., Valeric acid glyceride esters in feed promote broiler performance and reduce the incidence of necrotic enteritis. Poult Sci, 2018. 97(7): p. 2303-2311.
143. Zhao, L. and L. Zheng, A Review on Bioactive Anthraquinone and Derivatives as the Regulators for ROS. Molecules, 2023. 28(24).
144. Berillo, D., M. Kozhahmetova, and L. Lebedeva, Overview of the Biological Activity of Anthraquinons and Flavanoids of the Plant Rumex Species. Molecules, 2022. 27(4).
145. Feng, S. and W. Wang, Bioactivities and Structure-Activity Relationships of Natural Tetrahydroanthraquinone Compounds: A Review. Front Pharmacol, 2020. 11: p. 799.
146. Wang, X., et al., Synthesis and Antibacterial Activity Evaluation of Biphenyl and Dibenzofuran Derivatives as Potential Antimicrobial Agents against Antibiotic-Resistant Bacteria. Curr Issues Mol Biol, 2022. 44(9): p. 4087-4099.
147. Wang, Y., et al., Biphenyls in Clusiaceae: Isolation, structure diversity, synthesis and bioactivity. Front Chem, 2022. 10: p. 987009.
